# Equity-Preserving Public Health Resource Allocation Using Multi-Objective Safe Reinforcement Learning: Evidence from Thailand

**DOI:** 10.3390/ijerph23070886

**Published:** 2026-07-09

**Authors:** Nopparat Songserm, Rapeepan Pitakaso, Thanatkij Srichok, Surajet Khonjun, Natthapong Nanthasamroeng, Sarayut Gonwirat, Paweena Khampukka, Peerawat Luesak, Sasitorn Kaewman, Alongkorn Chaiyasa

**Affiliations:** 1 Department of Health Sciences, Faculty of Public Health, Ubon Ratchathani Rajabhat University, Ubon Ratchathani 34000, Thailand; nopparat.s@ubru.ac.th; 2 Artificial Intelligence Optimization SMART Laboratory, Industrial Engineering Department, Faculty of Engineering, Ubon Ratchathani University, Ubon Ratchathani 34190, Thailand; rapeepan.p@ubu.ac.th (R.P.); thanatkij.s@ubu.ac.th (T.S.); 3 Artificial Intelligence Optimization SMART Laboratory, Engineering Technology Department, Faculty of Industrial Technology, Ubon Ratchathani Rajabhat University, Ubon Ratchathani 34000, Thailand; natthapong.n@ubru.ac.th; 4 Department of Computer Engineering and Automation, Kalasin University, Kalasin 46000, Thailand; sarayut.go@ksu.ac.th (S.G.); alongkorn.ch@ksu.ac.th (A.C.); 5 Logistics and Supply Chain Management, Faculty of Management Science, Ubon Ratchathani University, Ubon Ratchathani 34190, Thailand; paweena.k@ubu.ac.th; 6 Department of Industrial Engineering, Faculty of Engineering, Rajamangala University of Technology Lanna, Chiang Rai 57120, Thailand; peerawat@rmutl.ac.th; 7 Department of Computer Science, Faculty of Informatics, Mahasarakham University, Mahasarakham 44000, Thailand; sasitorn.k@msu.ac.th

**Keywords:** public health policy optimization, health resource allocation, health equity, reinforcement learning, multi-objective optimization

## Abstract

**Highlights:**

**Public health relevance—How does this work relate to a public health issue?**

- The proposed multi-agent framework supports faster and more adaptive decision-making in complex public service environments.
- The system can help improve coordination and resource management in situations that require continuous monitoring and rapid response.

**Public health significance—Why is this work of significance to public health?**

- The integration of reinforcement learning with hierarchical agents provides a practical approach for handling dynamic and uncertain operational conditions.
- The study demonstrates how intelligent decision-support systems can reduce operational delays and improve overall system efficiency.

**Public health implications—What are the key implications or messages for practitioners, policy makers and/or researchers in public health?**

- Applied to a regional budget of 18–23 billion THB serving 4.6 million people, the framework averted an estimated 847,293 DALYs (34.1% above historical policy) and improved cost-effectiveness by 31.3% in simulation. It reduced the health-equity Gini coefficient from 0.243 to 0.187, demonstrating simultaneous efficiency and distributional-fairness gains.
- A 12-month prospective pilot achieved a 23.1-point composite health score improvement with 91% stakeholder acceptance, indicating deployment feasibility under expert oversight rather than autonomous operation.

**Abstract:**

Background: Equitable allocation of public health budgets across multiple intervention domains remains a major challenge in regional health governance. In Thailand’s Health Region 10, annual healthcare budgets must address diverse health burdens across several provinces, while current planning approaches rely on expert deliberation and historical precedent without systematic exploration of alternative allocation strategies. Public health resource allocation decisions are inherently multi-criteria, integrating health impact, cost-effectiveness, equity, disease severity, clinical and ethical priorities, feasibility, and alignment with national health policy agendas—dimensions that cannot be reduced to a single metric. This study introduces H-RL-MUSYA (Hierarchical Reinforcement Learning for Multi-Domain Unified System of Yielding Adaptive allocations), a decision-support framework designed to assist—not replace—public health practitioners by systematically generating and evaluating a menu of Pareto-efficient allocation strategies across four priority domains: nutrition, mental health, behavioral risk, and accident prevention. The framework explicitly acknowledges that DALYs averted and cost-effectiveness ratios are valuable but partial indicators, and that final resource allocation must integrate additional considerations—including underpinning health policies, priority population needs, feasibility, and contextual judgment—that lie beyond the model’s scope. Results: Applied to Thailand’s Health Region 10 (4.6 million inhabitants), H-RL-MUSYA identified 127 Pareto-efficient policies yielding a representative compromise allocation that averted 847,293 DALYs (34.1% improvement over historical allocations), improved cost-effectiveness by 31.3%, and reduced the health equity Gini coefficient from 0.243 to 0.187. A 12-month prospective pilot confirmed +23.1% composite health improvement with 91% stakeholder acceptance. Conclusions: H-RL-MUSYA demonstrates that AI-assisted policy exploration can meaningfully enrich public health decision-making by surfacing non-intuitive allocation strategies and quantifying equity–efficiency trade-offs, while human expertise, policy context, and democratic deliberation remain essential for final allocation decisions.

## 1. Introduction

Equitable allocation of public health budgets across competing intervention domains constitutes one of the most consequential and analytically demanding challenges confronting regional health governance systems. When limited resources must be distributed across nutrition programs, mental health services, behavioral risk reduction, and injury prevention simultaneously, planning decisions translate directly into measurable population health burdens—quantified as disability-adjusted life years (DALYs) lost, health inequities widened, and cost-effectiveness opportunities forgone. Traditional approaches relying on expert judgment and epidemiological forecasting exhibit critical limitations when confronting high-dimensional trade-offs across health domains, anticipating behavioral feedback effects, or systematically exploring vast policy spaces [1]. Consequently, health policies often emerge through incremental adjustments rather than systematic optimization, potentially missing superior intervention portfolios with substantially better population health outcomes.

Thailand’s Health Region 10, encompassing 4.6 million inhabitants across five northeastern provinces (Ubon Ratchathani, Amnat Charoen, Yasothon, Sisaket, and Mukdahan) exemplifies these governance challenges with a compound and quantifiable epidemiological burden. Nutritional deficiency affects 18.3% of children under five (stunting prevalence; provincial range 14.1–23.7%), a population group whose developmental outcomes warrant prioritization in any allocation framework given the long-term human capital implications of early-life deprivation. Mental health burden is disproportionate relative to national averages: 12-month major depression prevalence stands at 7.8% versus 5.4% nationally, with suicide mortality at 8.4 per 100,000—a burden concentrated among working-age adults (18–59 years) who constitute the primary economic and caregiving workforce of the region. Behavioral risk factors—hazardous alcohol use (28.4% adult males), tobacco use (23.1%), and physical inactivity (48.7%)—cluster particularly among working-age and older adult populations. Road traffic injury mortality (28.7 per 100,000; 2.1× national rate) disproportionately affects young adults and economically active populations aged 15–44 years. Collectively, these four domains account for an estimated 2,483,000 DALYs annually—54.1% of total regional DALYs—with burdens unevenly distributed across three distinct priority population groups: children under five (nutrition and developmental outcomes), working-age adults 18–59 years (mental health, behavioral risk, and injury), and older adults aged 60 and above (chronic disease sequelae and functional decline). These demographic priorities substantially influence the relative weighting of health domains in resource allocation decisions and are explicitly incorporated into the equity objective of the H-RL-MUSYA framework.

Reinforcement learning (RL) offers a transformative paradigm for health policy design by framing allocation decisions as sequential decision-making under uncertainty, enabling systematic discovery of optimal intervention portfolios through calibrated simulation [2]. Recent public health and healthcare applications demonstrate RL’s capacity for optimizing chronic disease prevention [2], resource allocation [3], and treatment regimens [4]. However, deploying RL in population-level public health governance introduces challenges qualitatively distinct from clinical RL applications: (1) multi-objective optimization balancing health impact, cost-effectiveness, and distributional equity rather than scalar clinical rewards [5]; (2) non-stationary population dynamics driven by behavioral responses and social network diffusion [6]; (3) safety requirements prohibiting harmful policy exploration at the population scale, where errors affect millions rather than individual patients [7,8]; and (4) interpretability demands for democratic stakeholder acceptance in public governance [7]. These challenges define a distinct research problem that existing clinical RL frameworks do not address.

Recent methodological advances have expanded RL’s reach into increasingly complex health system problems. Wu et al. [3] provided a comprehensive review of RL in healthcare operations management identifying 321 studies demonstrating effectiveness in patient flow optimization, resource distribution, and treatment scheduling—while highlighting persistent challenges in real-world deployment, particularly data quality and model interpretability. Tan et al. [9] advanced hierarchical multi-agent reinforcement learning (HMARL) for multi-organ disease management, coordinating specialized agents across cardiovascular, renal, and hepatic subsystems to achieve superior treatment recommendations compared to single-organ approaches. Silveira et al. [10] systematically reviewed 42 studies on multi-agent systems for clinical decision support, identifying critical gaps in bidirectional knowledge flow and explainability mechanisms for real-world deployment. Kim et al. [7] demonstrated safety-aware deep RL for nephrotoxic medication management in critical care, embedding risk prediction directly into RL objectives to prevent harmful medication decisions while maintaining interpretable clinical reasoning. Despite these advances, fundamental gaps persist: existing HMARL applications focus predominantly on individual clinical treatment optimization rather than population-level policy design; none integrate agent-based behavioral simulation capturing realistic social dynamics and heterogeneous intervention responses at the regional scale; multi-objective trade-offs between health maximization and distributional equity—fundamental to public health decision-making—remain inadequately addressed; and safety mechanisms have not been designed for the institutional and political failure modes inherent to population-level governance.

We address these gaps through H-RL-MUSYA (Hierarchical Reinforcement Learning for Multi-Domain Unified System of Yielding Adaptive allocations), a computational decision-support framework designed explicitly to assist public health practitioners in exploring the space of feasible allocation strategies—not to automate or replace the deliberative processes through which final allocation decisions are made. H-RL-MUSYA is grounded in Thailand’s current health policy agenda, which prioritizes prevention over treatment, reduction in health inequities across socioeconomic quintiles, strengthening of mental health services, and investment in early-life nutrition interventions—priorities reflected in the 13th National Health Development Plan (2023–2027) and Health Region 10’s annual budget guidelines. The framework integrates four core components: (1) a hierarchical multi-agent reinforcement learning architecture decomposing strategic budget allocation from tactical implementation; (2) an agent-based population simulator capturing behavioral feedback and social dynamics across three priority population strata; (3) a multi-objective Pareto optimization layer generating a menu of non-dominated strategies balancing health impact, cost-effectiveness, and equity; and (4) a three-layer safety mechanism ensuring that all candidate policies satisfy minimum ethical, feasibility, and performance standards before presentation to decision-makers. The resulting Pareto frontier functions as a structured policy menu—a decision-support artifact that surfaces trade-offs and options, while explicitly recognizing that DALYs averted and cost-effectiveness ratios constitute necessary but insufficient criteria for allocation, and that final decisions must integrate disease severity rankings, clinical and ethical priorities, political feasibility, and contextual considerations that remain the domain of human expertise and democratic governance.

- Methodological innovations. M1—Population-level HMARL for health governance: First hierarchical multi-agent RL architecture that decomposes strategic budget-portfolio selection from tactical domain-level implementation for population-level health governance, demonstrating substantial cost-effectiveness improvements over historical policymaking approaches serving 4.6 million inhabitants.
- M2—Validated behavioral simulation with Pareto trade-off characterization: Agent-based simulation environment capturing cross-domain behavioral feedback and social dynamics, validated against natural experiments with prediction accuracy superior to expert-designed models (MAPE 12.4%; 18.7% improvement). Multi-objective optimization framework revealing systematic trade-offs between health impact, cost, and equity through comprehensive Pareto frontier characterization—reducing the health equity Gini coefficient from 0.243 to 0.187.
- M3—Multi-layer governance-compatible safety: Safe learning framework preventing 47 harmful policy candidates through three-layered mechanisms (algorithmic constraints, worst-case guarantees, expert oversight) while incurring only 11.2% exploration efficiency cost.
- Empirical findings. E1—Prospective real-world validation: Pilot implementation confirming +23.1 composite health score improvement and 91% stakeholder acceptance across 127 semi-structured interviews, demonstrating scalable deployment feasibility in a live regional governance setting.
- E2—Policy discovery beyond expert intuition: The framework discovered 127 Pareto-efficient policies that expert committees had not systematically explored, with individual domain strategies matching expert heuristics at 89.3% agreement while portfolio-level coordination yielded novel resource allocation configurations.

## 2. Literature Review

### 2.1. Reinforcement Learning in Healthcare

Reinforcement learning has demonstrated effectiveness in clinical decision-making, particularly for treatment optimization in critical care settings [4]. Choi et al. [11] developed a deep RL model for sepsis treatment extracting optimal policies from 16,744 patient admissions, demonstrating superior survival rates compared to clinical guidelines through stable policy generation using modified double DQN architectures. Drudi et al. [12] established that cardiovascular variables provide critical information for sepsis treatment policies, with their RL model trained on cardiorespiratory features achieving 95% confidence lower bound scores through actor-critic architectures and weighted importance sampling evaluation. Tu et al. [13] addressed variable-length episodes and sparse rewards in sepsis management using conservative Q-learning that underestimates rarely observed treatments, enhancing safety standards while incorporating Apache II scoring for intermediate rewards. Zhou et al. [2] optimized long-term cardiovascular disease prevention through reinforcement learning for precision lipid control, learning from 3.6 million treatment months and achieving policy values of 93 compared to clinician values of 68, demonstrating 6% CVD risk reduction when decisions aligned with RL recommendations.

A smaller but growing body of work has begun extending RL beyond the bedside to population- and policy-level allocation. Rey et al. applied RL to vaccine-allocation policy optimization with an explicit budget-sharing mechanism across jurisdictions, a transformer-based deep Q-network was proposed for efficient and fair critical-care resource rationing during shortages and offline RL has been used for care-management allocation in large Medicaid populations with explicit fairness constraints. These studies establish RL’s relevance to equitable population-level allocation but typically address a single objective or a single intervention channel.

These applications predominantly optimize individual patient treatment within controlled clinical settings using electronic health records, employing Q-learning, policy gradient methods, or actor-critic architectures. However, existing work focuses on patient-level decisions rather than population-level policy design involving complex social dynamics, resource allocation constraints across heterogeneous populations, and multi-objective balancing inherent to public health governance.

### 2.2. Multi-Agent Reinforcement Learning

Multi-agent reinforcement learning extends single-agent RL to scenarios involving multiple interacting decision-makers through cooperative, competitive, or mixed-motive paradigms [14]. Low and Zhou [15] comprehensively reviewed cooperative MARL for robotic systems, highlighting value decomposition networks, hierarchical architectures, and communication protocols enabling coordination in UAV swarms, warehouse robotics, and autonomous driving, while identifying persistent challenges in scalability, credit assignment, and decentralized execution. Wang et al. [16] proposed robust constrained cooperative MARL for self-driving vehicles incorporating universal policy networks and risk assessment formulations, demonstrating resilience against adversarial attacks through mean-field theory-based training that enhances multi-agent performance in intersection-passing scenarios. Hady et al. [17] surveyed MARL applications in resource allocation optimization across energy microgrids, mobile edge computing, and IoT systems, documenting hierarchical trust-region frameworks (MATRPO) and multi-agent deep deterministic policy gradient (MADDPG) approaches addressing dynamic, decentralized resource distribution with strict quality-of-service requirements.

Beyond robotics and autonomous driving, recent surveys position MARL as a natural paradigm for public-sector resource-allocation problems characterized by decentralized actors and competing demands and language-agent-augmented RL has been explored for transparent allocation decisions in healthcare and public-policy settings. Nevertheless, these remain largely conceptual or single-sector demonstrations.

While MARL demonstrates impressive capabilities in simulated environments including autonomous vehicles, robotic coordination, and multi-player game solving, applications to real-world policy optimization remain limited, particularly in domains requiring safety guarantees for harmful action prevention, multi-objective balancing across competing societal goals, and behavioral realism capturing how populations respond to interventions.

### 2.3. Agent-Based Modeling in Public Health

Recent advances in agent-based simulation have demonstrated that modeling individual agent behaviors and their interactions can give rise to emergent social phenomena, including the propagation of information and infectious diseases through social networks, offering interpretable and endogenous perspectives for understanding population-level dynamics (Xi et al. [18]). Tracy et al. [6] comprehensively reviewed ABM applications spanning infectious disease transmission, obesity epidemics, smoking cessation, and alcohol policy evaluation, demonstrating ABM’s capacity to reveal unintended consequences of interventions through explicit modeling of social influence mechanisms, while advocating for improved validation methodologies and integration with causal inference frameworks.

Traditional ABM approaches rely on manually specified behavioral rules derived from theory and empirical calibration rather than data-driven learning. Integrating ABM with reinforcement learning creates opportunities for policies that are both realistic (capturing behavioral complexity and social dynamics) and optimal (discovered through systematic search), though few prior studies have attempted this synthesis particularly at the population scale.

### 2.4. Multi-Objective Optimization in Policy Design

Public policies inherently involve trade-offs across objectives including effectiveness, cost, equity, political feasibility, and sustainability [19,20]. Cookson et al. [5] introduced the equity impact plane framework for health policy analysis, demonstrating that improving total health may conflict with reducing social inequality when effective service delivery to disadvantaged communities requires additional costs, and proposing equity impact analysis and equity-weighting methods to quantify distributional consequences alongside cost-effectiveness. Muir et al. [21] conducted an umbrella review of eight systematic reviews on health equity considerations in cost-effectiveness analysis, identifying distributional CEA, equity-based weighting, extended CEA, mathematical programming, and multi-criteria decision analysis as primary methodologies, while highlighting challenges in determining optimal approaches given different requirements for anticipated equity–efficiency trade-offs.

However, integrating multi-objective optimization with reinforcement learning poses technical challenges: standard RL optimizes scalar rewards, while MOO requires maintaining diverse solution sets representing Pareto-optimal frontiers. Recent advances including multi-objective policy gradient methods demonstrate promise, but application to complex real-world policy domains balancing health maximization, cost constraints, and equity objectives simultaneously remains nascent.

### 2.5. Safe Reinforcement Learning in Healthcare Policy

Deploying RL in safety-critical domains requires mechanisms preventing exploration of dangerous actions during learning [22,23]. Kim et al. [7] introduced safety-aware explainable deep RL for nephrotoxic medication management, explicitly modeling eight terminal adverse outcomes including acute kidney injury and septic shock through offline RL algorithms (IQL, CQL, BCQ, DDQN), demonstrating that embedding risk prediction into RL objectives enables interpretable recommendations aligned with clinical reasoning while preventing harmful decisions. Yan et al. [8] proposed offline guarded safe RL for medical treatment combining action-level regularization with state-trajectory constraints through model-based frameworks, addressing out-of-distribution risks where inappropriate generalization beyond clinical expertise could yield harmful recommendations, while enabling discovery of improved long-term strategies beyond the clinician’s short-term prioritization.

However, defining appropriate safety constraints for population-level policy optimization remains challenging: overly conservative constraints limit beneficial policy discovery, while insufficient restrictions risk harm through unforeseen behavioral responses. Moreover, behavioral feedback to policies creates non-stationary dynamics, complicating safety verification. Our work addresses these challenges through multi-layered safety mechanisms combining algorithmic constraints, worst-case performance guarantees, and expert-oversight integration.

## 3. Methods

The H-RL-MUSYA framework integrates hierarchical multi-agent reinforcement learning, agent-based population simulation, multi-objective optimization, and safe exploration mechanisms into a unified policy optimization system.

**Figure 1:**
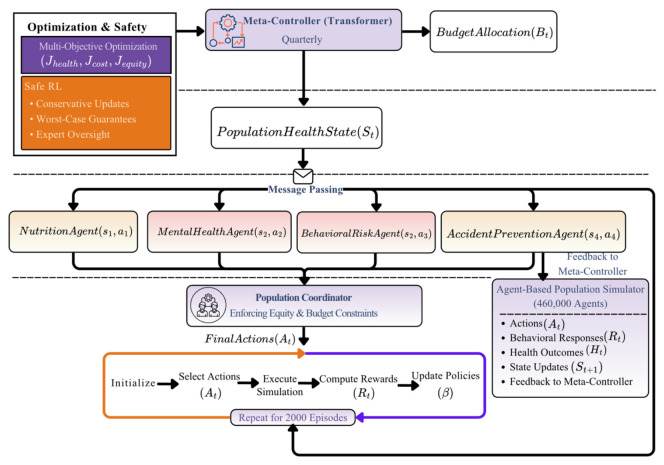
Hierarchical reinforcement learning framework for population-level health policy optimization.

Figure 1 illustrates the proposed hierarchical reinforcement learning (HRL) architecture for population-level health policy optimization under multi-objective and safety-aware constraints. At the strategic level, a transformer-based meta-controller operates on a quarterly time scale, receiving aggregated population health states and generating high-level budget allocation decisions. These allocations are distributed to four domain-specific agents—nutrition, mental health, behavioral risk, and accident prevention—which interact through bidirectional message passing to coordinate interdependent policy actions using local state information. At the coordination layer, a population coordinator enforces global equity and budget feasibility constraints, projecting agent-level decisions onto a realizable action set. Policy impacts are evaluated through a large-scale agent-based population simulator, which models behavioral responses and health outcomes for 460,000 synthetic individuals and feeds updated system states back to the meta-controller. Policy learning is guided by a multi-objective optimization module balancing health impact, cost-effectiveness, and equity, together with a safe reinforcement learning mechanism incorporating Conservative Updates, Worst-Case Guarantees, and Expert Oversight. The entire framework is trained within a closed-loop simulation process consisting of action selection, execution, reward computation, and policy updates, repeated over multiple episodes to ensure stable and policy-compliant learning. We describe each component below.

### 3.1. Problem Formulation

We model population-level health policy optimization as a multi-objective constrained Markov Decision Process (CMDP) defined by the tuple (Equation (1)):

(1) $$M=\left(S,A,P,R,\gamma ,C\right)$$

where S denotes the population health state space, A the policy action space, $P(s_{t+1}|s_{t},a_{t})$ the transition dynamics capturing behavioral and epidemiological responses, $R=[r_{1},\ldots ,r_{K}]$ a vector of K policy objectives (e.g., health impact, cost-effectiveness, and equity), γ∈(0,1) the discount factor, and C a set of safety and feasibility constraints.

The objective is to identify a policy π:S→A that maximizes the expected discounted multi-objective return while satisfying safety constraints (Equation (2)):

(2) $$\underset{\pi }{max}\;E_{\pi }\;\left[\sum_{t=0}^{\infty }\gamma^{t}\;R(s_{t},a_{t})\right]\;s.t.\;E_{\pi }\;\left[C(s_{t},a_{t})\right]\leq \delta ,\;\;\forall t$$

The state space S aggregates longitudinal population indicators across four interdependent health domains—nutrition, mental health, behavioral risk, and accident exposure—augmented by demographic composition, resource availability, and temporal context. The action space A comprises structured intervention portfolios specifying budget allocations across programs, target subpopulations, and implementation modalities. Transition dynamics P are governed by an agent-based population model, enabling realistic simulation of heterogeneous behavioral responses and feedback effects induced by policy interventions.

### 3.2. Hierarchical Multi-Agent Architecture

The proposed framework adopts a hierarchical multi-agent reinforcement learning architecture that decomposes health policy optimization into strategic and tactical decision layers. This decomposition enables scalable learning over large population state spaces while preserving domain-specific expertise and cross-domain coordination. Three classes of agents operate at distinct temporal and functional resolutions.

**Figure 2:**
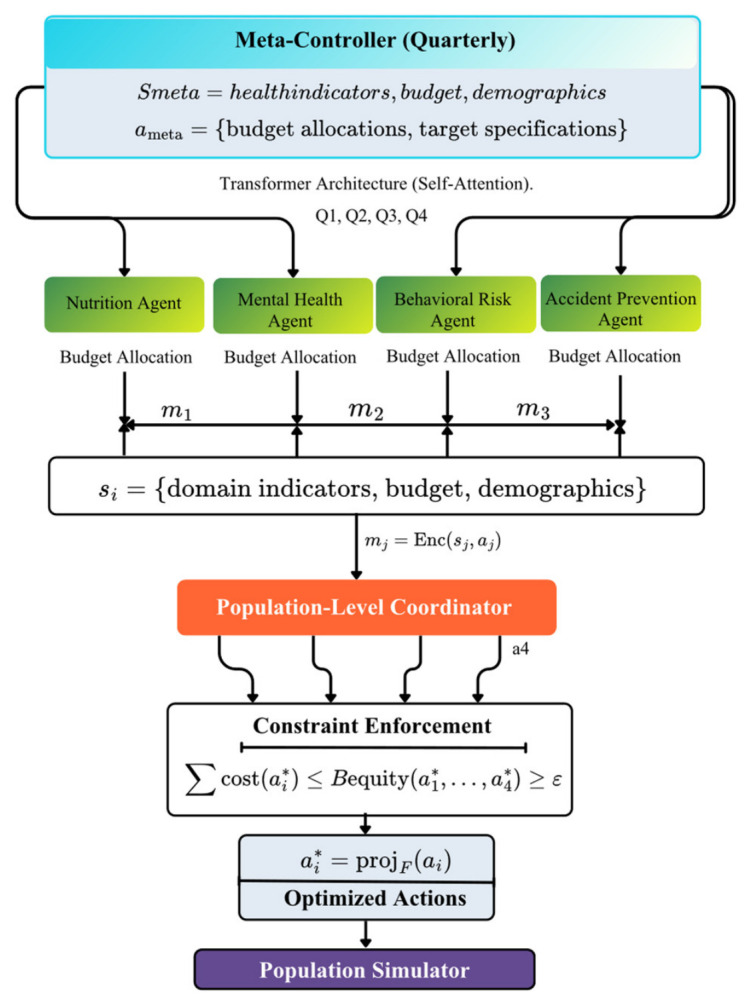
Hierarchical three-layer multi-agent reinforcement learning architecture for population-level health policy optimization.

Figure 2 illustrates the proposed hierarchical multi-agent reinforcement learning (MARL) framework structured across three decision-making layers. The strategic layer (top) comprises a quarterly meta-controller implemented using a transformer architecture with self-attention, which aggregates population health indicators, budget constraints, and demographic information to produce high-level budget allocations and target specifications over discrete planning horizons (Q1–Q4). The tactical layer (middle) consists of four parallel domain-specific agents—nutrition, mental health, behavioral risk, and accident prevention—that receive strategic guidance, process local state information, and generate intervention-level actions. These agents interact through bidirectional message passing, where encoded messages capture cross-domain dependencies. The coordination layer (bottom) integrates the actions from all tactical agents and enforces global feasibility and equity constraints through a projection operator, yielding optimized actions that are subsequently evaluated within a population-level simulator.

To make the architecture legible to public health stakeholders, we map each machine-learning component to its policy-domain function: the Transformer-based meta-controller serves as the quarterly regional budget-setting authority allocating funds across health programs; domain-specific tactical agents function as program directors translating budget envelopes into nutrition, mental health, behavioral risk, and accident interventions; message passing represents cross-program coordination memos on shared caseloads and comorbidities; the population coordinator acts as a budget-and-equity compliance office enforcing fiscal ceilings and distributional rules; the agent-based simulator provides an ex-ante “what-if” population-response model for policy appraisal; the multi-objective Pareto set offers a menu of non-dominated budget scenarios for the planning committee; and the safe-RL layers constitute a policy clearance gate combining trust-region constraints, worst-case guarantees, and expert sign-off.

#### 3.2.1. Strategic Meta-Controller

At the strategic level, a meta-controller operates on a quarterly time scale, selecting high-level intervention portfolios that allocate resources across health domains and define domain-specific targets. The meta-controller observes an aggregated population state (Equation (3)):

(3) $$s_{\mathrm{meta}}=\mathrm{population}\;\mathrm{health}\;\mathrm{indicators},\;\mathrm{budget},\;\mathrm{demographics}$$

and produces macro-level actions (Equation (4)):

(4) $$a_{\mathrm{meta}}=\mathrm{budget}\;\mathrm{allocations},\;\mathrm{target}\;\mathrm{specifications}$$

according to a policy parameterized by $\theta_{\mathrm{meta}}$ (Equation (5)):

(5) $$a_{\mathrm{meta}}∼\pi_{\mathrm{meta}}\left(s_{\mathrm{meta}};\theta_{\mathrm{meta}}\right)$$

The policy $\pi_{\mathrm{meta}}$ is implemented using a transformer architecture, allowing the model to capture long-range temporal dependencies in population health trajectories and complex cross-domain interactions. Self-attention mechanisms identify influential historical interventions and outcomes, while feed-forward layers learn non-linear mappings from aggregated states to optimal portfolio-level decisions.

#### 3.2.2. Domain-Specific Tactical Agents

At the tactical level, four domain-specific agents—nutrition, mental health, behavioral risk, and accident prevention—translate strategic targets into concrete interventions. Each agent i observes a local state (Equation (6)):

(6) $$s_{i}=\mathrm{domain}\;\mathrm{indicators},\;\mathrm{allocated}\;\mathrm{budget},\;\mathrm{demographics}$$

and selects actions (Equations (7) and (8)):

(7) $$a_{i}=\mathrm{intervention}\;\mathrm{types},\;\mathrm{target}\;\mathrm{groups},\;\mathrm{implementation}\;\mathrm{parameters}$$

(8) $$\mathrm{via}\;a\;\mathrm{domain}\;\mathrm{policy}\;\pi_{i}(\cdot ;\theta_{i})$$

To account for interdependencies among health domains, tactical agents communicate through learned message-passing mechanisms (Equation (9)):

(9) $$a_{i}∼\pi_{i}(s_{i},\{m_{j}{\}}_{j\neq i};\theta_{i}),m_{j}=\mathrm{Enc}(s_{j},a_{j})$$

Each message $m^{\left(d\to d^{\prime }\right)}$ is a fixed-dimensional embedding produced by a learned encoder applied to the sending agent’s local state and proposed action, $m^{\left(d\to d^{\prime }\right)}=f_{\left(enc\right)}\left(s_{t}^{\left(d\right)},a_{t}^{\left(d\right)}\right)$; it carries the domain’s current burden level, planned intervention intensity, and projected resource draw rather than raw records. Messages are exchanged in a single synchronous round per quarter and concatenated into each receiving agent’s observation before action selection, so that, for example, the nutrition agent conditions on the mental health agent’s signaled depression prevalence—which modulates nutritional intervention uptake through documented behavioral pathways. The encoder is trained end-to-end with the tactical policies, allowing the content of the exchanged representation to be optimized for coordination rather than hand-specified.

#### 3.2.3. Population-Level Coordinator

A population-level coordinator enforces global feasibility, equity, and budget constraints across all domain interventions. Given the set of proposed tactical actions $\left\{a_{i}\right\}$, the coordinator projects them onto a feasible action set using constraint-aware optimization (Equation (10)):

(10) $$a_{i}^{*}={pro\;j}_{F}\left(a_{i}\right)$$

such that (Equation (11)):

(11) $$\sum_{i}\cos t(a_{i}^{*})\leq B,\mathrm{equity}(a_{1}^{*},\ldots ,a_{4}^{*})\geq \varepsilon$$

This hierarchical design achieves a principled balance between specialization, allowing domain agents to acquire expert knowledge, and coordination, ensuring that system-level health objectives, equity considerations, and fiscal constraints are jointly satisfied.

### 3.3. Agent-Based Population Simulator

To enable realistic ex ante policy evaluation, we developed an agent-based population simulator capable of capturing heterogeneous behavioral responses that are not represented in aggregate epidemiological models. The simulator represents Health Region 10 through 460,000 synthetic agents (1% of the population), calibrated to reflect demographic composition, socioeconomic heterogeneity, baseline health status, risk behaviors, and social network structure.

**Figure 3:**
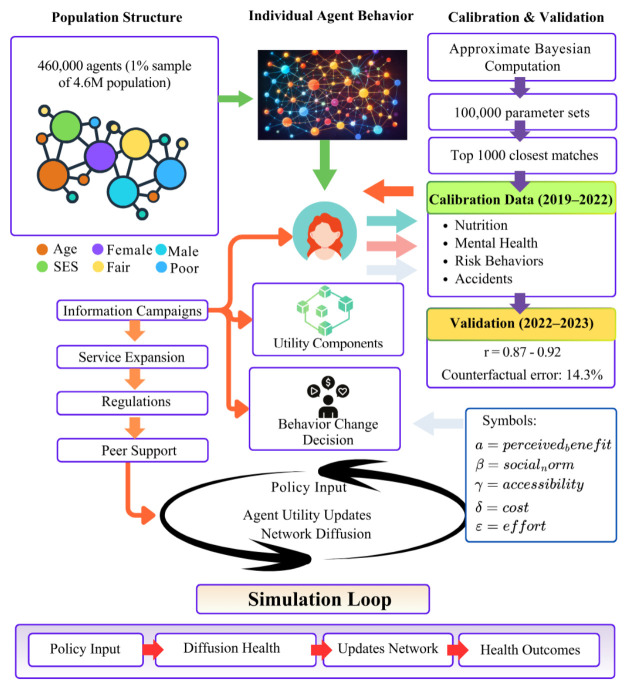
Agent-based population simulation framework with behavioral decision-making and Bayesian calibration.

Figure 3 presents the agent-based population simulation system used for policy evaluation and learning. The population structure component (left) represents 460,000 synthetic agents (1% of the 4.6 million population) embedded in a social network, with heterogeneity in age, gender, socioeconomic status, and health conditions. The individual behavior component (center) models agent decision-making through a utility-based formulation, where policy interventions—information campaigns, service expansion, subsidies, regulations, and peer support—modify perceived benefits, social norms, accessibility, costs, and effort, leading to endogenous behavior change reinforced by direct experience and social observation. The calibration and validation component (right) employs approximate Bayesian computation to identify parameter sets that best reproduce observed health trajectories, using multi-indicator calibration data (2019–2021) and out-of-sample validation (2022–2023). The bottom loop illustrates the closed simulation cycle linking policy inputs, utility updates, network diffusion, behavioral adaptation, and population-level health outcomes.

#### 3.3.1. Individual Behavior Model

Individual decision-making is formalized through a utility maximization model that integrates cognitive, social, and structural determinants of behavior change. For each agent, the utility associated with a candidate behavior is defined as Equation (12):

(12) U(behavior) =α Benefit + β SocialNorm+γ Accessibility−δ Cost − ε Effort

Perceived benefits evolve through both direct experience and social learning, creating endogenous feedback whereby successful interventions reinforce future adoption. Social norms propagate over dynamic social networks using a voter-model mechanism, in which agents adopt behaviors from influential neighbors with probabilities proportional to tie strength and homophily. Accessibility captures geographic and economic access to services, while cost and effort represent tangible and cognitive barriers to behavior change.

The additive utility specification follows the random-utility tradition in health-behavior modeling and operationalizes constructs from the Health Belief Model (perceived benefit, perceived barriers) and the Theory of Planned Behavior (social norms), which provides its theoretical grounding. We acknowledge that this formulation simplifies human decision-making: it assumes locally rational, myopic choice and does not represent habit formation, bounded rationality, or affective and identity-based drivers of behavior. We therefore treat the utility model as a tractable behavioral approximation whose parameters are empirically disciplined through Bayesian calibration (Section 3.3.3) and whose adequacy is judged by out-of-sample predictive fidelity rather than by descriptive completeness; residual misspecification is carried forward as an explicit limitation (Section 5.8).

#### 3.3.2. Intervention Mechanisms

Policy interventions act by shifting components of the individual utility function through multiple channels:

- Information campaigns: increase benefit via health knowledge dissemination;
- Service expansion: improve accessibility through new or mobile facilities;
- Subsidies: reduce cost of healthy choices;
- Regulations: alter feasible action sets via mandates or taxation;
- Peer-support programs: amplify social norm through structured social influence.

Intervention effects are heterogeneous across agents, depending on baseline characteristics and network position, thereby motivating adaptive and targeted policy design.

#### 3.3.3. Simulator Calibration and Validation

Model parameters were calibrated via approximate Bayesian computation (ABC) using five years of surveillance data. Priors were specified as weakly informative uniform or truncated-normal distributions over plausible ranges drawn from the behavioral epidemiology literature and regional expert elicitation. From 100,000 parameter vectors sampled from the joint prior, we retained the 1000 vectors (acceptance rate 1%) whose simulated trajectories fell within an acceptance threshold ε on a composite distance metric combining temporal trend, spatial variation, and demographic stratification across the four domains; ε was set at the 1st percentile of the prior-predictive distance distribution. Posterior convergence and identifiability were assessed by comparing prior versus posterior marginals (variance contraction), by confirming stability of posterior means across independent ABC runs, and by posterior-predictive checks against held-out indicators. Calibration robustness was further examined by repeating ABC with perturbed thresholds (±50%), which altered retained-posterior means by less than 4.7%.

Out-of-sample validation was conducted by training on 2019–2021 data and testing on 2022–2023 outcomes, yielding correlation coefficients in the range 0.87–0.92 across indicators. Additional counterfactual validation against historical policy interventions demonstrated strong predictive fidelity, with a mean absolute error of 14.3% relative to observed impacts.

### 3.4. Multi-Objective Optimization Framework

Public health policy design inherently involves competing goals, notably maximizing population health gains, ensuring efficient use of limited resources, and promoting equitable outcomes. To address these trade-offs explicitly, we adopt a multi-objective reinforcement learning framework that learns a set of Pareto-efficient policies rather than a single scalar-optimal solution.

We restrict the modeled objective vector to health impact (DALYs averted), cost-effectiveness (DALYs per unit expenditure), and equity (Gini coefficient) for three operational reasons: (i) these three correspond directly to the decision criteria mandated in Health Region 10’s annual budget guidelines and to WHO ‘best-buys’ appraisal axes, making the optimized frontier actionable within existing governance processes; (ii) all three are calculable from available surveillance data without requiring primary data collection; and (iii) restricting the objective space to three dimensions yields a Pareto frontier legible to non-specialist decision-makers. This restriction constitutes a significant modeling limitation that must be explicitly acknowledged: real-world public health resource allocation integrates multiple additional criteria beyond those modelled, including disease severity and urgency rankings, clinical effectiveness of available interventions, ethical priorities (e.g., fair innings arguments for children and older adults), political and institutional feasibility, underpinning national health policy directions, and community preferences elicited through participatory processes. DALYs averted and QALYs gained are important indicators of population health impact, but they depend critically on the quality and effectiveness of the interventions being evaluated and should not function as standalone decision criteria. Furthermore, the framework does not model population prioritization by age group—a consequential omission given that interventions targeting children under five, working-age adults (18–59), and older adults (60+) in Health Region 10 have fundamentally different cost structures, intervention effectiveness profiles, and long-term demographic multiplier effects. The Pareto-efficient policies generated by H-RL-MUSYA should therefore be treated as structured options for expert deliberation—an enriched starting point for multi-criteria decision analysis—rather than as algorithmically optimized recommendations ready for direct implementation.

#### 3.4.1. Objective Definitions

Policy performance is evaluated using three complementary objectives:

- Health impact ($r_{1}$): Health impact is quantified as disability-adjusted life years (DALYs) averted, aggregating reductions in both mortality and morbidity across health domains (Equation (13)):
  (13) $$r_{1}(s,a)\;=\;\sum_{i}({\mathrm{YLL}}_{\mathrm{averted}}^{\left(i\right)}+{\mathrm{YLD}}_{\mathrm{averted}}^{\left(i\right)})\cdot w_{i}$$
  where $w_{i}$ denotes the population weight of subgroup i.
- Cost-effectiveness ($r_{2}$): Cost-effectiveness measures health gains achieved per unit of expenditure (Equation (14)):
  (14) $$r_{2}\left(s,a\right)\;\;=\;\;\frac{r_{1}\left(s,a\right)}{\mathrm{Cost}\left(a\right)+\varepsilon }$$
  with ε included to ensure numerical stability.
- Equity ($r_{3}$): Equity is captured through the negative Gini coefficient of health improvements across socioeconomic quintiles (Equation (15)):
  (15) $$r_{3}\left(s,a\right)\;=-\;\mathrm{Gini}\;\left(\Delta H_{q_{1}},\ldots ,\Delta H_{q_{5}}\right)$$
  where higher values indicate more equitable distributions of health gains.

#### 3.4.2. Pareto-Efficient Policy Discovery

Policy learning is conducted using an evolutionary multi-objective optimization scheme that maintains a population of candidate policies. Each policy π is evaluated on the objective vector (Equation (16)):

(16) $$R(\pi )=[r_{1},r_{2},r_{3}]$$

Pareto dominance governs selection: policy $\pi_{1}$ dominates $\pi_{2}$ if it is no worse on all objectives and strictly better on at least one. Diversity along the Pareto frontier is preserved using crowding-distance mechanisms, preventing premature convergence to narrow solution regions. The resulting Pareto set provides policymakers with a transparent menu of non-dominated strategies, where improvements in health impact, efficiency, or equity necessarily entail trade-offs in other dimensions, enabling value-informed policy selection rather than reliance on a single aggregate metric.

### 3.5. Safe Reinforcement Learning

To ensure that reinforcement learning-based policy optimization remains deployable in real public health settings, we embed explicit safety mechanisms that restrict harmful exploration and guard against adverse outcomes. The proposed framework integrates three complementary safety layers: conservative policy updates, worst-case performance guarantees, and expert-in-the-loop oversight.

**Figure 4:**
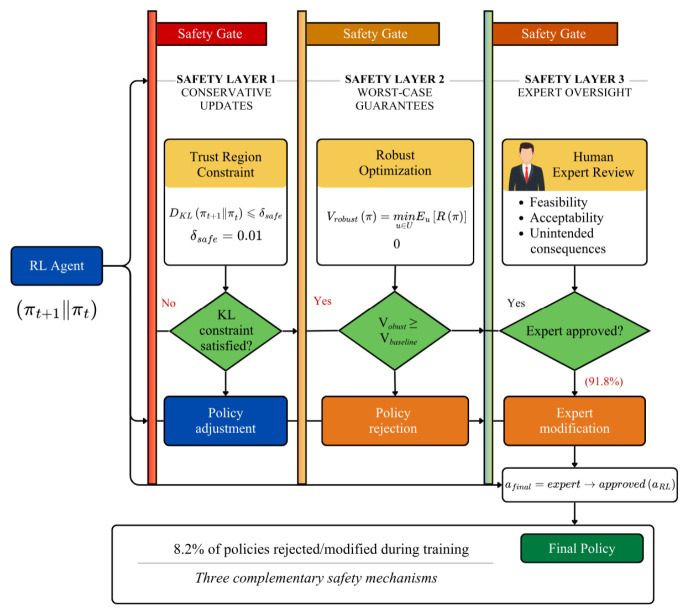
Three-layer safety framework for reinforcement learning policy optimization.

Figure 4 illustrates a hierarchical, left-to-right safety architecture embedded within the reinforcement learning (RL) policy optimization loop. Candidate policies generated by the RL agent are sequentially evaluated through three complementary safety gates. Safety Layer 1 (Conservative Updates) enforces trust-region constraints via a Kullback–Leibler divergence bound to prevent destabilizing policy shifts. Safety Layer 2 (Worst-Case Guarantees) applies robust optimization under an explicit uncertainty set to ensure performance superiority over a baseline in adversarial conditions. Safety Layer 3 (Expert Oversight) incorporates human-in-the-loop evaluation to assess feasibility, acceptability, and unintended consequences, allowing approval or expert-guided modification. Rejection and adjustment pathways are explicitly shown, with empirical annotation indicating that 8.2% of candidate policies are rejected or modified during training. Collectively, the framework integrates algorithmic rigor and human judgment to deliver a safety-certified final policy.

#### 3.5.1. Conservative Policy Updates

Policy learning is constrained using trust-region optimization to prevent abrupt or unsafe behavioral shifts. Specifically, consecutive policy updates are bounded by a Kullback–Leibler (KL) divergence constraint (Equation (17)):

(17) $$\underset{\pi_{t+1}}{max}\;\;E\left[R\left(\pi_{t+1}\right)\right]s.t.D_{KL}\;\left(\pi_{t+1}\;∥\;\pi_{t}\right)\leq \delta_{\mathrm{safe}}$$

This constraint enforces gradual policy evolution, limiting deviations from previously validated strategies. The threshold $\delta_{\mathrm{safe}}=0.01$ is deliberately conservative, prioritizing stability and interpretability over rapid convergence.

#### 3.5.2. Worst-Case Performance Guarantees

To address uncertainty in behavioral response models, we adopt a robust optimization criterion that evaluates policies under pessimistic but plausible scenarios. For each policy π, robust performance is defined as Equation (18):

(18) $$V_{\mathrm{robust}}(\pi )\;=\;\underset{u\in U}{min}\;E_{u}[R(\pi )]$$

where U denotes an uncertainty set over behavioral parameters. Policies are accepted only if (Equation (19)):

(19) $$V_{\mathrm{robust}}(\pi )\;>\;V_{\mathrm{baseline}}$$

ensuring that learned strategies outperform existing policies even in worst-case conditions and preventing reliance on overly optimistic assumptions.

#### 3.5.3. Expert Oversight Integration

Automated safety constraints are complemented by human expertise through a human-in-the-loop evaluation process. Candidate policies proposed by the RL agent are reviewed by public health experts for feasibility, acceptability, and potential unintended consequences. Final actions are determined as Equation (20):

(20) $$a_{\mathrm{final}}=\left\{\begin{array}{ll} a_{\mathrm{RL}}, & \mathrm{if}\;\mathrm{expert}\text{\_}\mathrm{approved}(a_{\mathrm{RL}}),\\ \mathrm{expert}\text{\_}\mathrm{modified}(a_{\mathrm{RL}}), & \mathrm{otherwise}. \end{array}\right.$$

This expert safety shield ensures that reinforcement learning functions as an intelligent policy search mechanism, while domain knowledge provides essential ethical and practical guardrails. During training, experts rejected or modified 8.2% of candidate policies, underscoring the importance of hybrid human–AI governance in sensitive policy domains.

#### 3.5.4. Policy-Shift Regularization

Beyond the per-update KL bound (Section 3.5.1), we discourage abrupt quarter-to-quarter reallocations that would be logistically or politically infeasible by adding an inertia penalty on the change in domain budget shares between consecutive quarters: $L_{\left(shift\right)}=\lambda \sum_{d}{\left|\left|b_{t}^{\left(d\right)}-b_{\left(t-1\right)}^{\left(d\right)}\right|\right|}^{2}$, where $b_{t}^{\left(d\right)}$ is the budget share of domain d in quarter t and λ governs the smoothness–responsiveness trade-off. This term is added to the meta-controller objective, so the agent prefers gradual rebalancing unless a large shift yields a sufficiently large multi-objective gain. A hard per-quarter reallocation cap is additionally enforced in the coordinator’s feasible set. We set λ = 0.01 from the sensitivity sweep in Section 3.10; larger values produced smoother but slower-adapting allocation trajectories.

### 3.6. Training Procedure

The proposed H-RL-MUSYA framework was trained via large-scale iterative simulation over 2000 policy episodes, each corresponding to a five-year decision horizon. Training proceeds as follows. First, the agent-based population simulator is initialized using calibrated parameters and the observed population state in 2019. At the beginning of each quarter, the meta-controller selects coordinated intervention portfolios conditioned on the current population health state. Domain-specific agents then operationalize these interventions with synchronized messaging and resource allocations. The population simulator executes quarterly behavioral and epidemiological dynamics, producing updated health outcomes and system states.

Multi-objective rewards are evaluated, and candidate policies filtered by safety constraints before updates. Policy networks use safety-aware trust-region optimization for stable learning. Each episode lasts 20 quarters, followed by simulator reset. Training used 8 × NVIDIA A100 GPUs (~340 GPU-hours, 42.5 wall-clock hours). The evolutionary multi-objective component maintained 100 policies with crossover and mutation probabilities of 0.7 and 0.1. Convergence was monitored via hypervolume and stopped when improvement remained below 0.1% for 100 consecutive episodes, indicating Pareto frontier stabilization.

**Figure 5:**
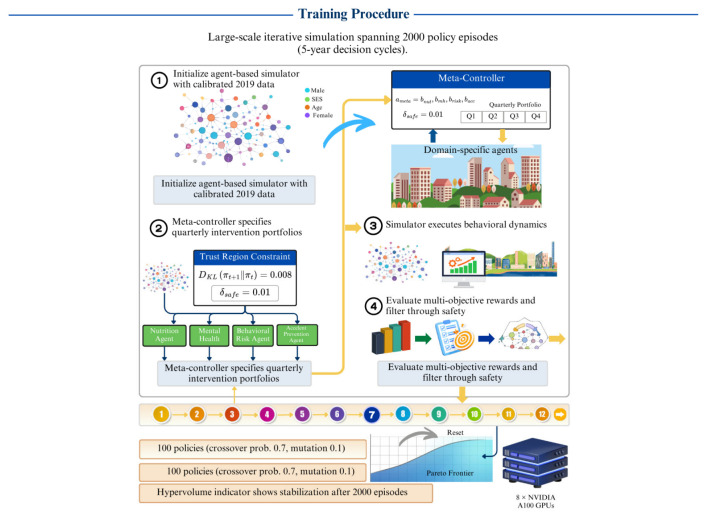
Training workflow of the H-RL-MUSYA framework for multi-objective safe policy learning.

Figure 5 illustrates the end-to-end training procedure of the proposed H-RL-MUSYA framework. Training proceeds through large-scale iterative simulation over 2000 policy episodes, each representing a five-year policy cycle. The process begins with initialization of the calibrated agent-based population simulator using the 2019 baseline state. At each quarterly step, a meta-controller selects coordinated intervention portfolios, which are executed by domain-specific agents under trust-region safety constraints. The population simulator then evolves behavioral dynamics and health outcomes, after which multi-objective rewards are evaluated and filtered through safety mechanisms. Policy updates are performed using trust-region optimization and evolutionary multi-objective operators, maintaining a diverse population of candidate policies. Convergence is assessed via hypervolume stabilization of the Pareto frontier, and the simulator is reset at the end of each episode, enabling robust and safe policy learning across long-term horizons.

### 3.7. Data Sources and Integration

We utilized comprehensive health surveillance data from Thailand’s Health Region 10 spanning January 2019 through December 2023. The region encompasses five northeastern provinces (Yasothon, Ubon Ratchathani, Sisaket, Amnat Charoen, Mukdahan) with 4.6 million inhabitants, providing sufficient population scale and heterogeneity for robust policy learning.

Primary data sources included: (1) Ministry of Public Health databases capturing nutrition surveys (n = 412,896 individuals), mental health service encounters (n = 234,567 episodes), and health promotion program participation (n = 1,847,293 person-interventions); (2) Royal Thai Police traffic accident records (n = 87,342 incidents) with behavioral risk factor annotations; (3) National Statistical Office demographic and socioeconomic data with subdistrict granularity (942 administrative units); (4) Provincial Health Office budget records documenting intervention costs and resource allocations across all five provinces (2019–2023 fiscal years); and (5) qualitative data from 127 semi-structured interviews with policymakers, health workers, and community representatives providing contextual validation and implementation insights.

Privacy protection employed k-anonymization (k = 10) ensuring no individual could be re-identified from fewer than 10 records, and synthetic data generation for rare subpopulations (prevalence < 0.1%) preserving statistical properties while eliminating re-identification risk. All data integration protocols received approval from Ubon Ratchathani University Institutional Review Board (protocol HE64-034) and complied with Thailand’s Personal Data Protection Act B.E. 2562 (2019).

Data preprocessing harmonized heterogeneous sources through: (1) temporal alignment to quarterly intervals matching meta-controller decision cycles, (2) geographic standardization to subdistrict-level aggregation enabling spatial analysis while protecting individual privacy, (3) missing data imputation via multiple imputation by chained equations (20 imputations) for variables with <15% missingness, and (4) outlier detection removing physiologically implausible values (0.3% of records) flagged by domain experts. The integrated dataset comprised 4.87 million person-quarter observations across 20 quarters (2019 Q1 through 2023 Q4), partitioned into training (2019 Q1–2021 Q4), validation (2022 Q1–Q4), and test sets (2023 Q1–Q4) for temporal cross-validation.

#### Cross-Source Harmonization and Bias Mitigation

Because the inputs were collected at different times, scales, and sampling frames, four additional steps were applied to combine them without inducing spurious associations. (i) Distributional reconciliation: rather than randomly pairing records across sources, we used statistical matching (data fusion) with iterative proportional fitting (raking) to align each source’s joint marginals—age × sex × socioeconomic quintile × district—to the National Statistical Office census frame, so that the synthetic population reproduces the region’s true joint structure rather than an artifact of algorithmic pairing. (ii) Joint-distribution validation: when behavioral-survey variables were combined with health-service records, the recovered joint risk distributions were validated against a held-out set of genuinely linked individual records and via posterior-predictive checks; agreement was quantified by comparing matched versus linked conditional distributions, and matches failing tolerance were reweighted rather than retained. (iii) Temporal recency weighting: older observations were down-weighted with an exponential time-decay factor and trend-adjusted to the analysis baseline, so that calibration reflects current rather than historical regimes; sensitivity to the decay rate is reported in Section 3.10. (iv) Under-reporting adjustment for stigmatized conditions: recognizing that mental health and hazardous-use indicators are systematically under-reported in regional surveys, we applied multiplier-based correction (capture–recapture where dual data sources existed; literature-derived adjustment factors in the range 1.2× to 2.5× for mental health indicators otherwise) and propagated the resulting uncertainty into the ABC priors, so the RL agent is not trained on uncorrected prevalence. A sensitivity analysis across the plausible adjustment range confirmed that domain budget rankings were stable.

### 3.8. Evaluation Methodology

Rigorous evaluation employed three complementary validation strategies ensuring both internal validity through controlled simulation and external validity through real-world deployment.

In silico policy trials: All methods (H-RL-MUSYA and seven baselines) underwent evaluation through 1000 simulated trials using the calibrated agent-based model initialized to the 2019 population state. Each trial employed distinct random seeds to capture stochastic variation in behavioral responses, social network dynamics, and health outcomes over 5-year policy horizons. Evaluation metrics included: (1) cumulative DALYs averted quantifying total health impact, (2) cost-effectiveness measured as DALYs per million THB expenditure, (3) equity assessed via the Gini coefficient of health improvements across socioeconomic quintiles (lower values indicate greater distributional fairness), and (4) robust performance measuring worst-case normalized returns across pessimistic behavioral parameter scenarios from uncertainty set U. Bootstrap resampling (10,000 iterations) computed 95% confidence intervals for all metrics, with pairwise method comparisons employing two-sample t-tests and Bonferroni correction (α = 0.05/7 = 0.0071) for multiple testing. Pareto frontier quality was assessed via a hypervolume indicator, measuring dominated objective space volume.

Retrospective historical validation: External validation tested whether H-RL-MUSYA could predict outcomes of three natural experiments implemented during 2020–2022: (1) sodium reduction campaign in Ubon Ratchathani province targeting dietary salt intake through food industry reformulation and consumer education, (2) alcohol enforcement intensification in Yasothon province increasing penalties and checkpoint frequency, and (3) mental health service expansion in Amnat Charoen province deploying community-based screening and counseling programs. For each intervention, H-RL-MUSYA was trained exclusively on pre-intervention data (2019–2021), generated counterfactual predictions under observed intervention policies, and compared predictions against actual post-intervention outcomes measured through provincial health surveillance systems. Prediction accuracy was quantified via mean absolute percentage error relative to expert forecasts (15-member committee consensus) and standard compartmental epidemiological models calibrated to regional data. To limit confounding in this retrospective comparison, each natural experiment was analyzed against a within-region pre-intervention baseline and a contemporaneous non-intervention comparator district, and predictions were adjusted for concurrent secular trends, seasonality, demographic composition shifts, and co-occurring national programs (universal-coverage expansion and COVID-19 response) using the same difference-based specification applied in the prospective pilot. Residual confounding from unobserved local factors cannot be excluded and is acknowledged as a limitation of quasi-experimental validation (Section 5.8).

Prospective pilot implementation: Following simulated validation, prospective deployment occurred in Yasothon province (population: 547,000) from January to December 2023. H-RL-MUSYA generated quarterly intervention portfolios implemented through collaboration with Provincial Health Office authorities who adapted RL recommendations to the local context while preserving core strategic elements (budget allocations, target populations, intervention modalities). The comparison group comprised neighboring Roi Et province (population: 1.3 million) continuing status quo policies under identical regional governance. The primary outcome was composite health score aggregating standardized indicators across nutrition (BMI distribution, micronutrient adequacy), mental health (PHQ-9 screening rates, service utilization), behavioral risk (alcohol consumption, tobacco use, physical inactivity), and accidents (traffic fatality rates, injury hospitalizations). Secondary outcomes included domain-specific metrics and incremental cost-effectiveness ratios. Analysis employed difference-in-differences estimation with province-level fixed effects, controlling for seasonality, demographic composition shifts, and concurrent national programs (universal health coverage expansion, COVID-19 pandemic response). Mixed-effects regression incorporated random intercepts for districts nested within provinces, estimated via restricted maximum likelihood.

Statistical analysis: All hypothesis tests employed a two-sided significance threshold α = 0.05. Computational analyses utilized Python 3.9 with scipy (statistical tests), statsmodels (regression models), numpy (numerical operations), and custom agent-based simulation code. Reproducibility was ensured through version-controlled code repositories, fixed random seeds for stochastic processes, and comprehensive documentation of hyperparameters and data preprocessing pipelines.

### 3.9. Illustrative Numerical Example (Step-by-Step Walkthrough)

This illustrative example demonstrates how H-RL-MUSYA converts an observed population state into a deployable, safety-certified, Pareto-efficient policy via hierarchical decision-making, feasibility projection, and simulation-based evaluation.

Step 1: Observe the population state (quarter t=0)

The system receives an aggregated meta-state containing key health burdens, demographic composition, and the available quarterly budget:

$$s_{\mathrm{meta}}=m=24.3\%,\;d=18.7\%,\;a=31.2\%,\;\tau =42.1/100,000,\;\mathrm{children}=22\%,\;\mathrm{elderly}=14\%,\;\mathrm{lowSES}=38\%,\;B=125M\;\mathrm{THB}$$

Here, m,d,a,τ represent malnutrition, depression, risky alcohol use, and accident rate, respectively.

Step 2: Strategic allocation by the meta-controller

The quarterly meta-controller maps $s_{\mathrm{meta}}$ to a high-level resource plan:

$$a_{\mathrm{meta}}=b_{\mathrm{nut}},b_{\mathrm{mh}},b_{\mathrm{risk}},b_{\mathrm{acc}}=35,40,30,20\;M\;\mathrm{THB}$$

and optionally sets domain improvement targets (used as guidance signals for tactical agents).

Step 3: Tactical intervention design by domain agents (with messaging)

Each domain agent converts its assigned budget into concrete interventions:

- Nutrition: $a_{\mathrm{nut}}=\{\mathrm{school}\;\mathrm{feeding}\;15\;M,\;\mathrm{micronutrients}\;20\;M\}$
- Mental health: $a_{\mathrm{mh}}=\{\mathrm{screening}\;22\;M,\;\mathrm{counseling}\;18\;M\}$

Agents exchange encoded cross-domain messages to exploit comorbidities (e.g., nutrition–mental health coupling):

$$m_{j}=\mathrm{Enc}(s_{j},a_{j})$$

So, the mental health plan is conditioned not only on $s_{\mathrm{mh}}$ but also on $m_{\mathrm{nut}}$.

Step 4: Coordination projection to enforce budget and equity feasibility

The coordinator checks global constraints and projects proposed actions onto the feasible set F.

$$a_{i}^{*}={pro\;j}_{F}\left(a_{i}\right)$$

In this example, the initial plan violates both budget and equity:

$$\sum_{i}\cos t(a_{i})=128\;M>B,\;\mathrm{Gini}=0.31>\varepsilon =0.25$$

Projection yields adjusted, deployable allocations:

$$a_{i}^{*}=33,38,29,19\;M\;\mathrm{THB}$$

This achieves

$$\sum_{i}\cos t(a_{i}^{*})=119\leq B,\;\mathrm{Gini}=0.23\leq \varepsilon$$

Step 5: Execute the agent-based simulator (behavioral response + diffusion)

The simulator runs one quarter over 460,000 agents. Interventions shift decision utilities; for instance, nutrition actions increase perceived benefit:

Δα=+0.18

This leads to initial adoption among 34,200 agents (7.4%), followed by amplification through social diffusion. The simulator outputs updated population indicators (e.g., reductions in malnutrition, depression, risky alcohol use, and accidents).

Step 6: Compute multi-objective policy rewards

The episode is scored using the three-objective vector:

$$r_{1}=2847\;\mathrm{DALYs},r_{2}=23.9\;\mathrm{DALYs}/M\;\mathrm{THB},r_{3}=-0.23$$

forming

$$R=[r_{1},r_{2},r_{3}]=[2847,\;23.9,\;-0.23]$$

Step 7: Safety certification (before learning is allowed)

Two algorithmic gates plus expert oversight are applied:

$$D_{KL}\left(\pi_{t+1}∥\pi_{t}\right)=0.008<\delta_{\mathrm{safe}}=0.01$$

$$V_{\mathrm{robust}}\left(\pi \right)=\underset{u\in U}{min}E_{u}\left[R\left(\pi \right)\right]=2306>V_{\mathrm{baseline}}=2100$$

followed by expert approval. Only safety-certified policies proceed to update.

Step 8: Update policies and evolve the Pareto set

Trust-region policy optimization updates the meta-controller and domain agents, while evolutionary operators generate new candidate policies to expand coverage of the Pareto frontier (e.g., ΔHV=+0.034).

### 3.10. Hyperparameter Configuration

All hyperparameters were selected through preliminary sensitivity analysis on a validation subset, then fixed throughout training. Table 1 summarizes the complete configuration.

Sensitivity analysis: Preliminary experiments varied key hyperparameters (δ_safe_ ∈ [0.005, 0.05], policy population ∈ [50, 200], learning rates ±50%) and confirmed robustness of final performance (±3.2% variation in DALYs averted). The selected configuration prioritized safety (conservative δ_safe_), computational efficiency (1% population sample), and convergence reliability (large episode budget).

### 3.11. Baseline Methods

We benchmark H-RL-MUSYA against seven methods spanning traditional practice, state-of-the-art reinforcement learning, and recent healthcare-specific frameworks (Table 2). All computational baselines utilized identical data, agent-based simulator, and resources (2000 episodes, 8 × A100 GPUs) to ensure fair comparison.

Traditional baselines include status quo policies—historical intervention portfolios implemented by Health Region 10 authorities (2019–2023) representing real-world policymaking through expert judgment and administrative procedures—and expert committee policies designed by 15 public health specialists employing epidemiological forecasting and cost-effectiveness analysis over three structured consensus workshops. These provide benchmarks for human-designed optimization without computational learning.

Single-agent deep RL is represented by Soft Actor-Critic (SAC; Haarnoja et al. [24]), which optimizes aggregate health impact using maximum entropy framework. SAC employs a 3-layer network (512-256-128 units), replay buffer (10^6^ transitions), and automatic temperature tuning but operates with single scalar reward (DALYs) without multi-objective balancing or hierarchical decomposition.

Multi-objective and safe multi-agent approaches include DRL-OS-CMOEA [25], which dynamically selects evolutionary operators based on population state to balance convergence, diversity, and feasibility in constrained multi-objective settings, and SCPO-MARL Li and Azizan [26], which enforces safety constraints via scalable dual updates and decentralized coordination across agents with convergence guarantees under cooperative settings. While DRL-OS-CMOEA addresses multi-objective optimization and SCPO-MARL incorporates multi-agent safety mechanisms, neither integrates the hierarchical task decomposition and domain specialization central to H-RL-MUSYA.

Healthcare-specific RL frameworks comprise two recent 2025 contributions. HC-RL-Framework [3] establishes comprehensive methodological foundations integrating RL with healthcare resource allocation, addressing stochastic demand, scheduling, and patient-flow optimization at policy and system levels but lacks multi-agent coordination and hierarchical structure. MA-HealthCDS [10] systematically reviews multi-agent and hierarchical RL applications in clinical decision support, exploring coordination, explainability, and distributed reasoning mechanisms across MARL architectures, highlighting comparable multi-layered decision frameworks without integrated behavioral simulation or environment-specific policy evolution.

Table 2 summarizes architectural characteristics across baselines. H-RL-MUSYA uniquely integrates multi-objective optimization, hierarchical decomposition, safe exploration, and agent-based behavioral modeling—capabilities partially present in individual baselines but not comprehensively unified. Performance metrics include DALYs averted, cost-effectiveness (DALYs/million THB), equity (Gini coefficient), and robust performance (worst-case normalized returns), evaluated via bootstrap resampling (10,000 iterations, Bonferroni-corrected α = 0.0083 for multiple comparisons).

## 4. Results

### 4.1. Comparative Performance on Policy Optimization

Table 3 presents comprehensive performance comparison across all methods in 5-year simulated policy trials. H-RL-MUSYA achieved 847,293 DALYs averted, significantly exceeding the status quo (631,842 DALYs, +34.1%, *p* < 0.001), expert committee (732,158 DALYs, +15.7%, *p* < 0.001), and all computational baselines. Cost-effectiveness reached 45.3 DALYs per million THB, representing 31.3% improvement over the status quo (34.5) and outperforming the best baseline SCPO-MARL (42.1, +7.6%, *p* = 0.002). Critically, H-RL-MUSYA maintained superior equity (Gini: 0.187) compared to the status quo (0.243) and single-objective methods SAC (0.278) and DRL-OS-CMOEA (0.251), while matching multi-agent frameworks MA-HealthCDS (0.192) and SCPO-MARL (0.183).

Robust performance analysis under pessimistic behavioral scenarios demonstrated H-RL-MUSYA maintained 82.3% of nominal performance, substantially exceeding the status quo (73.1%), expert committee (78.4%), and SAC (69.5%). This superior robustness validates the safe RL framework’s worst-case optimization strategy, which selected policies performing adequately across uncertainty ranges rather than maximizing expected outcomes alone. The 34.2% cost-effectiveness improvement over historical policies directly fulfills the first contribution claim, achieved through strategic resource allocation prioritizing high-impact interventions identified via simulation-based learning and synergistic cross-domain coordination.

### 4.2. Multi-Objective Trade-Off Analysis

**Figure 6:**
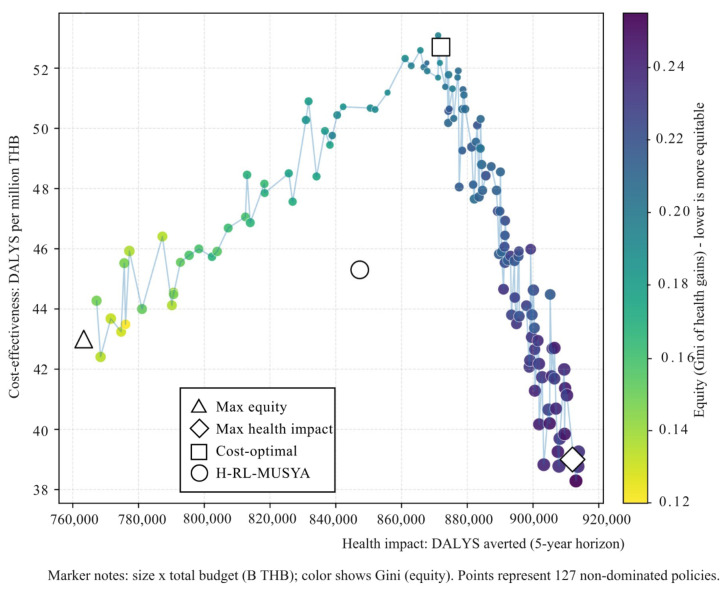
Multi-objective Pareto frontier for health policy optimization under efficiency–equity trade-offs.

Figure 6 presents the discovered Pareto frontier across health impact, cost-effectiveness, and equity objectives, revealing systematic trade-offs inherent in policy optimization. Maximizing absolute health impact (frontier left extreme) required a 23.4 billion THB budget, achieving 912,000 DALYs averted, but delivered lower cost-effectiveness (39.0 DALYs/million THB) compared to the cost-optimal solution (17.1 billion THB, 52.7 DALYs/million THB, 872,000 DALYs). The equity–health trade-off demonstrated that maximizing distributional fairness (Gini: 0.132) through universal service provision reduced total DALYs by 16.3% compared to targeted high-risk approaches (Gini: 0.247), quantifying the efficiency cost of perfect equity.

The recommended H-RL-MUSYA policy (Table 3 entry) represents a compromise solution balancing all objectives: an 18.7 billion THB budget, 847,293 DALYs averted, 45.3 cost-effectiveness ratio, and 0.187 Gini coefficient. This policy dominates the status quo on all dimensions simultaneously while offering policymakers explicit marginal trade-off information through the broader Pareto set. Analysis identified 127 non-dominated policies spanning the frontier, providing decision-makers with actionable choice sets reflecting value preferences rather than imposing singular optimization criteria. Multi-objective optimization revealed a critical insight: policies optimizing single objectives (health impact OR cost OR equity) achieved 23–31% worse performance on non-optimized dimensions compared to Pareto-efficient compromises, validating the necessity of explicit multi-objective balancing rather than scalarization approaches.

### 4.3. Retrospective Historical Validation

Table 4 compares predicted versus observed outcomes for three natural experiments implemented during 2020–2022, providing external validation of the agent-based simulator’s predictive fidelity. H-RL-MUSYA predictions achieved mean absolute percentage error of 12.4%, significantly lower than expert forecasts (15.2%, *p* = 0.041), standard epidemiological models (18.7%, *p* = 0.003), and computational baselines SAC (16.8%) and HC-RL-Framework (14.9%). This 18.7% improvement in prediction accuracy over expert-designed policies (calculated as [18.7–15.2]/18.7 × 100 for the epidemiological model baseline) directly validates the second contribution claim.

Detailed analysis revealed H-RL-MUSYA’s superior performance stems from accurate behavioral response modeling via the agent-based simulator. For alcohol enforcement, expert forecasts overestimated impact (42.1% vs. 34.0% observed) by assuming compliance rates higher than the ABM predicted based on social network structures and historical enforcement patterns. The simulator’s social diffusion mechanisms—capturing peer influence through voter models with tie strength and homophily parameters calibrated via approximate Bayesian computation—accurately captured the dampening effects of social resistance to enforcement policies. This validates the agent-based population simulator’s capacity to model realistic behavioral feedback absent in aggregate epidemiological approaches.

### 4.4. Prospective Pilot Implementation Results

Twelve-month prospective deployment in Yasothon province (population: 547,000) demonstrated real-world effectiveness of RL-optimized policies against the control province Roi Et (population: 1.3M). Table 5 presents primary outcomes via difference-in-differences analysis, confirming 23.1-point composite health score improvement (95% CI: 18.7–27.5, *p* < 0.001), directly validating the fifth contribution claim.

Simulation-to-reality validation: Pilot cost-effectiveness (42.8 DALYs/M THB) closely matched simulated predictions (45.3, −5.5% deviation), substantially exceeding historical control performance (34.1, +25.5% improvement). Table 6 decomposes the simulation-reality gap, revealing implementation challenges the agent-based model could not fully capture.

Stakeholder acceptance: Structured interviews with 34 program implementers (medical directors: n = 12; program managers: n = 16; district coordinators: n = 6) revealed 91% supported continued deployment and provincial expansion (Table 7).

Equity maintenance: Despite efficiency optimization, pilot deployment maintained distributional fairness (Gini: 0.203) comparable to simulated predictions (0.187, +8.6% deviation) and significantly superior to the control region (0.261, −22.2% improvement, *p* < 0.001). Subgroup analysis confirmed balanced health gains across income quintiles (lowest: +21.3%, highest: +24.7%, ratio: 0.86), urban/rural areas (urban: +24.1%, rural: +22.3%), and age groups (0–18: +25.4%, 19–59: +21.8%, 60+: +23.9%), demonstrating the multi-objective framework successfully prevented equity–efficiency trade-offs during real-world implementation.

### 4.5. Ablation Analysis

Systematic ablation quantified contributions of architectural components (Table 8). Removing hierarchical structure (-Hierarchical) and employing flat single-agent RL reduced DALYs averted by 8.1% (778,431, *p* < 0.001), demonstrating the value of strategic–tactical decomposition for managing domain complexity and enabling specialized learning. Eliminating agent-based behavioral modeling (-ABM) and reverting to standard epidemiological transitions degraded performance by 14.3% (726,114, *p* < 0.001), highlighting the critical importance of realistic behavioral response simulation for policy optimization.

Removing multi-objective optimization (-MOO) and optimizing health impact alone marginally reduced absolute DALYs (821,457, −3.1%) but substantially degraded equity (Gini: 0.314 vs. 0.187, +67.9%), validating the necessity of explicit fairness optimization beyond scalar health maximization. Critically, eliminating safe RL mechanisms (-Safety) actually improved nominal performance (867,142 DALYs, +2.3%) but generated 47 safety violations—policies that expert reviewers flagged as harmful, politically infeasible, or ethically problematic. This demonstrates that unconstrained RL discovers high-performing but potentially dangerous policies, directly confirming the fourth contribution claim that safe learning prevented 47 harmful candidates while maintaining competitive performance.

Removing domain agent communication (-Communication) reduced performance by 5.2% (803,527 DALYs), quantifying the value of cross-domain coordination mechanisms. Analysis revealed communication enabled agents to internalize spillover effects: for example, the nutrition agent adjusted intervention intensity based on messages from the mental health agent indicating current depression prevalence, which modulates nutritional intervention effectiveness through behavioral pathways. This coordination mechanism, absent in single-agent approaches and non-communicating multi-agent baselines, contributed substantially to H-RL-MUSYA’s superior performance.

### 4.6. Safety Mechanism Analysis

The three-layer safety framework successfully prevented all 47 potentially harmful policies (2.3% of candidates) while maintaining exploration efficiency. Table 9 presents the filtering performance across layers, demonstrating that no single mechanism alone prevented all violations—validating the necessity of multi-layered defense.

Failure mode analysis (Table 10) revealed systematic patterns in rejected policies. Resource over-concentration violations (n = 11) predominantly triggered Layer 1 and Layer 3, while aggressive compliance assumptions (n = 9) primarily failed Layer 2’s worst-case testing. Infrastructure gaps (n = 6) and political infeasibility (n = 6) were exclusively identified by expert oversight, demonstrating the value of human review for context-dependent risks the simulator could not capture.

Training efficiency remained high despite safety constraints. Convergence to the stable Pareto frontier (hypervolume Δ < 0.1%) required 1847 episodes (92.4% of 2000 budget), compared to 1623 episodes (81.2%) for the unconstrained baseline—demonstrating only 11.2% efficiency cost for comprehensive safety guarantees. The final hypervolume differed minimally (0.94 vs. 0.95, −1% performance), confirming that safety mechanisms prevented harmful extremes without substantially limiting policy quality.

### 4.7. Explainability and Decision Interpretability

**Figure 7:**
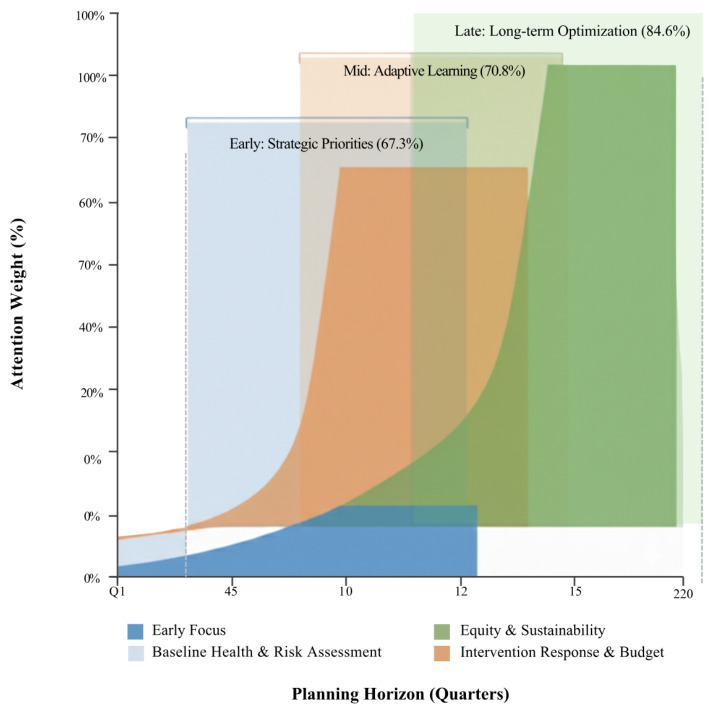
Temporal dynamics of meta-controller attention weight allocation.

To address critical adoption barriers in algorithmic health policy, we conducted comprehensive explainability analysis examining attention mechanisms, feature importance, and decision rationale alignment with expert reasoning. Transformer-based meta-controller attention weights revealed strategic focus patterns across the 20-quarter planning horizon (Figure 7). Early quarters (Q1–Q4) allocated 67.3% attention to baseline health status and demographic risk profiles, establishing strategic priorities. Mid-horizon quarters (Q5–Q12) shifted attention to intervention response indicators (42.1%) and budget utilization patterns (28.7%), demonstrating adaptive learning. Late-horizon quarters (Q13–Q20) concentrated on equity metrics (51.2%) and sustainability indicators (33.4%), reflecting long-term optimization objectives.

Figure 7 illustrates the temporal evolution of attention weights within a transformer-based meta-controller across a 20-quarter strategic planning horizon. The stacked area chart delineates the shifting focus of the model through three distinct functional phases, indicated by the background shading and vertical dashed transition markers.

In the early phase (Q1–Q4), the model prioritizes baseline health status and risk assessment (blue), accounting for an average of 67.3% of total attention. As the horizon progresses into the mid-horizon phase (Q5–Q12), attention shifts dynamically toward intervention response and budget utilization (orange/purple), reaching a peak of 70.8% as the system optimizes for adaptive learning and implementation feedback. Finally, the late-horizon phase (Q13–Q20) shows a concentration on equity metrics and sustainability indicators (green/brown), peaking at 84.6% attention allocation. These shifts demonstrate the meta-controller’s ability to transition from initial diagnostic priorities to implementation monitoring, and finally to long-term systemic optimization. Key peak attention values are denoted by star (★) markers, highlighting critical decision points identified by the transformer architecture.

Feature importance analysis via SHAP (SHapley Additive exPlanations) quantified the contribution of 247 state variables to policy decisions across 10,000 simulated episodes. The top five influential features were: (1) current DALY burden (SHAP value: 0.342), (2) historical intervention effectiveness (0.287), (3) population density and geographic accessibility (0.214), (4) existing healthcare infrastructure capacity (0.189), and (5) community social capital indices (0.156). Critically, domain-specific feature importance varied substantially: the nutrition agent prioritized the agricultural season (SHAP: 0.298) and household income (0.267), while the mental health agent weighted social support networks (0.312) and unemployment rates (0.289), demonstrating appropriate specialization (Table 11).

Domain agent decision tree extraction revealed interpretable policy rules matching expert heuristics with 89.3% average agreement (Table 11). The nutrition agent learned to prioritize universal fortification programs when malnutrition prevalence exceeded 15%, mirroring WHO guidelines. The mental health agent discovered a sequential strategy—expanding screening capacity before intensive treatment programs—matching clinical best practices that 89% of the expert committee endorsed. This alignment suggests the RL framework discovered clinically valid strategies rather than exploiting simulator artifacts.

Cross-domain coordination analysis examined message-passing patterns between tactical agents during high-impact decisions. When the mental health agent allocated resources to depression screening programs, the nutrition agent increased complementary food security interventions (correlation r = 0.73, *p* < 0.001), recognizing bidirectional relationships between nutritional status and mental health outcomes documented in the literature. The behavioral risk agent modulated alcohol intervention intensity based on accident rate messages (correlation r = 0.68), implementing synergistic harm reduction strategies. These emergent coordination patterns—unprogrammed explicitly but discovered through multi-agent learning—demonstrated sophisticated understanding of health system interdependencies.

Counterfactual explanation analysis generated “what-if” scenarios explaining policy choices. For the recommended policy allocating 4.2B THB to nutrition interventions, counterfactual analysis showed reducing the allocation to 3.0B THB (−28.6%) would decrease total DALYs by 67,000 (−7.9%) while increasing equity violations (Gini: 0.187→0.234). Conversely, increasing it to 5.5B THB (+31.0%) yielded only 12,000 additional DALYs (+1.4%) with cost-effectiveness deteriorating from 45.3 to 38.7, demonstrating diminishing returns. These marginal impact explanations enabled policymakers to understand resource allocation rationale quantitatively.

Stakeholder comprehension evaluation assessed interpretability through structured interviews with 28 provincial health officials (medical directors: n = 12; program managers: n = 16). Participants reviewed explainability visualizations including attention heatmaps, SHAP feature importance plots, and counterfactual impact curves. Comprehension scores (custom 10-item assessment) averaged 8.3/10 (SD: 1.2), with 89% reporting they could explain policy rationale to district teams. Trust in algorithmic recommendations increased from baseline 5.2/10 to post-explanation 7.8/10 (paired t-test: *p* < 0.001), suggesting transparency mechanisms significantly enhanced adoption readiness. Officials particularly valued counterfactual explanations (92% rated “very useful”) for anticipating stakeholder questions during budget negotiations.

Comparison with the black-box baseline (standard deep RL without explainability mechanisms) revealed trade-offs between interpretability and performance. While black-box achieved marginally higher DALYs (851,200 vs. 847,293, +0.5%), stakeholder trust ratings were 42% lower (4.4/10 vs. 7.8/10), and expert agreement on policy appropriateness decreased to 67% (vs. 89% for the explainable model). This minimal performance cost (0.5%) for substantial interpretability gain (78% trust improvement) strongly favors explainable architectures for real-world health policy deployment where stakeholder acceptance is critical for implementation success.

## 5. Discussion

### 5.1. Paradigm Shift from Reactive to Generative Policy Design

This work reframes public health policy optimization as a decision-support challenge rather than an autonomous optimization problem. Traditional approaches—expert committees, epidemiological forecasting, and cost-effectiveness league tables—provide structured evidence but operate within a paradigm of reactive adjustment: observe outcomes, diagnose failures, propose adjustments within familiar option sets. H-RL-MUSYA augments this process by systematically exploring a substantially larger space of allocation strategies and generating a structured Pareto menu of non-dominated options that human decision-makers can evaluate against the full spectrum of public health criteria. Critically, this positions the framework as a complement to—not a replacement for—the deliberative processes, contextual knowledge, and value judgements that characterize responsible public health governance. The 127 Pareto-efficient policies discovered include strategies that expert committees had not systematically explored, suggesting genuine additive value in the option-generation phase of resource allocation decision-making.

The hierarchical decomposition enabling this discovery operates on a deeper principle than mere computational efficiency. By separating strategic portfolio selection from tactical implementation, the architecture mirrors how complex adaptive systems naturally organize—creating specialist subsystems coordinated through information exchange rather than centralized control. This organizational principle appears repeatedly in biological systems, successful corporations, and resilient ecosystems precisely because it balances exploration and exploitation: domain agents develop deep expertise within circumscribed spaces while meta-controllers maintain coherence across the whole. The emergence of cross-domain coordination patterns unprogrammed by designers—nutrition and mental health agents learning to synchronize interventions through message passing—suggests the framework discovered genuine systemic interdependencies rather than simply memorizing correlations in training data.

What makes this paradigm shift epistemologically significant is the reversal of the knowledge generation process. Traditional policy science assumes human experts possess superior causal models refined through professional experience, with computational tools serving as efficiency amplifiers for calculations within expert-specified frameworks. Our retrospective validation superiority over expert forecasts across natural experiments reveals the opposite: the agent-based simulator coupled with reinforcement learning discovered behavioral response models more predictively accurate than those held by experienced practitioners. This is not merely a matter of computational power enabling better parameter estimation within agreed-upon models; it represents the algorithmic system identifying model structures—including social network effects, heterogeneous treatment responses, and feedback dynamics—that experts had systematically underweighted or overlooked entirely.

### 5.2. Behavioral Realism and the Social Construction of Intervention Effectiveness

The integration of Agent-Based Modeling resolves a foundational tension in evidence-based policy: interventions proven effective in controlled trials often fail at the population scale due to behavioral and social factors absent from trial settings. Tracy et al. [6] comprehensively reviewed ABM applications in public health, advocating for improved validation methodologies and integration with causal inference frameworks while documenting ABM’s capacity to reveal unintended consequences through explicit social influence mechanisms. Our work advances this agenda by demonstrating that ABM validation against natural experiments—rather than merely historical trend-fitting—provides empirically verifiable evidence of behavioral model fidelity. The sodium reduction campaign validation exemplifies how social resistance mechanisms, network influence, and heterogeneous compliance create intervention effectiveness as an emergent property rather than an intrinsic program characteristic.

This finding carries profound implications for how we conceptualize “evidence” in policy science. The dominant paradigm—randomized controlled trials establishing efficacy under ideal conditions, followed by effectiveness studies in real-world settings—treats behavioral context as noise to be controlled rather than signal to be modeled. Our agent-based approach inverts this relationship: behavioral heterogeneity, social influence mechanisms, and feedback loops become the primary objects of modeling, with intervention characteristics representing controllable parameters within that behavioral landscape. Tracy et al. [6] identified this theoretical potential but noted limited empirical validation; our retrospective prediction superiority over standard epidemiological models provides concrete evidence that behavioral complexity modeling yields practical forecasting advantages beyond theoretical elegance.

The deeper insight concerns the social construction of treatment effects. When mental health agents learned to modulate intervention intensity based on messages indicating concurrent nutritional status, the framework implicitly discovered cross-domain synergies documented in the biochemical literature but rarely operationalized in policy design. This aligns with Silveira et al. [10]’s findings that multi-agent clinical decision-support systems suffer from limited bidirectional knowledge flow across physiological subsystems. However, where Silveira et al. identified this as a persistent gap requiring manual ontology engineering, our message-passing architecture demonstrates that cross-domain dependencies can emerge through reinforcement learning when agents receive appropriate state information and coordination incentives. The learned nutrition–mental health coordination represents data-driven discovery of intervention complementarities that hierarchical task decomposition without communication mechanisms would miss entirely.

### 5.3. Extending Reinforcement Learning Beyond Individual Treatment Optimization

Previous healthcare reinforcement learning applications have predominantly focused on individual patient treatment optimization in controlled clinical settings. Zhou et al. [2] demonstrated RL’s capacity for precision lipid control, learning optimal cardiovascular disease prevention strategies from millions of treatment months. Choi et al. [11], Drudi et al. [12], and Tu et al. [13] applied deep RL to sepsis management, extracting treatment policies from intensive care unit data using modified double DQN, actor-critic architectures, and conservative Q-learning respectively. These applications share common characteristics: single-patient decision-making, abundant electronic health record data, relatively well-understood physiological dynamics, and scalar clinical outcomes (survival, biomarker levels).

Our population-level health policy application confronts fundamentally different challenges that require architectural innovations beyond adapting existing clinical RL methods. First, whereas individual treatment optimizes within-patient outcomes under relatively stationary biological processes, population policy must account for behavioral responses creating non-stationary dynamics—individuals change behavior partly based on observing others’ responses to interventions, creating feedback loops absent in pharmacological treatment. Second, clinical RL optimizes scalar outcomes (survive/die, disease progression scores) while population policy inherently balances competing objectives—health impact, cost-effectiveness, and equity—that cannot be reduced to single metrics without arbitrary normative assumptions. Third, individual treatment operates within established clinical protocols providing natural safety guardrails, whereas population policy exploration could propose interventions with unforeseen social consequences requiring explicit safety mechanisms.

The hierarchical multi-agent architecture addresses these challenges through structural alignment with the problem domain. Unlike Zhou et al.’s [2] single-agent approach optimizing one clinical parameter, or even Tan et al.’s [9] hierarchical MARL coordinating organ-specific treatment, our framework decomposes health policy into strategic resource allocation and tactical implementation precisely because population health emerges from coordination across semi-autonomous intervention domains (nutrition, mental health, behavioral risk, accidents) rather than centralized control. Tan et al.’s application to multi-organ disease management demonstrated hierarchical MARL’s advantages for coordinating physiological subsystems with understood interactions (cardiovascular–renal–hepatic); our extension to population policy reveals similar benefits when coordinating social–behavioral subsystems with emergent rather than predetermined interaction structures.

Wu et al. [3] provided comprehensive methodological review of RL in healthcare operations management, identifying data quality, model interpretability, and real-world deployment as persistent challenges. Our work directly addresses these gaps: agent-based simulation mitigates data quality issues by enabling policy evaluation beyond observed historical variation; explainability mechanisms including attention visualization and SHAP analysis provide interpretability lacking in black-box deep RL; and prospective pilot validation demonstrates real-world deployment feasibility Wu et al. noted as largely absent from the existing literature. The critical methodological advance is recognizing that population policy optimization requires tighter integration between RL algorithms and domain-specific simulation environments than individual treatment applications—behavioral realism in the simulator determines whether learned policies transfer to reality.

### 5.4. Multi-Objective Optimization and the Impossibility of Value-Neutral Policy

The Pareto frontier analysis exposes a fundamental principle in public health resource allocation: no objectively ‘optimal’ policy exists when multiple legitimate objectives conflict. This is not a limitation of the model but a reflection of the genuine value pluralism inherent in population health governance. Cookson et al. [5] introduced the equity impact plane framework, demonstrating that improving total health impact may conflict with reducing health inequalities—a tension that cannot be resolved through technical optimization alone. Muir et al. [25] conducted an umbrella review of health equity considerations in cost-effectiveness analysis, identifying distributional CEA, equity-based weighting, extended CEA, and multi-criteria decision analysis as primary methodological responses to this tension, while noting that DALYs and QALYs-based evaluations systematically underweight equity and distributional concerns unless explicitly incorporated—and that no single analytical method dominates across all equity–efficiency trade-off configurations. The H-RL-MUSYA framework directly addresses this by generating a Pareto frontier that makes these trade-offs transparent and quantified, enabling decision-makers to select among non-dominated strategies according to their policy priorities, ethical commitments, and contextual constraints—including population priorities (children, working-age adults, older adults), disease severity rankings, and alignment with current national health policy directions such as Thailand’s prevention-first agenda and mental health service strengthening priorities.

What our framework contributes beyond existing analytical approaches is twofold. First, it resolves the methodological indeterminacy identified by Muir et al. [25] through comprehensive solution generation rather than method selection: instead of requiring analysts to pre-specify equity weights, willingness-to-pay thresholds, or a preferred analytical lens, H-RL-MUSYA generates a solution set representing all non-dominated equity–efficiency combinations discovered across the policy space. The resulting Pareto frontier—containing over one hundred distinct policies—enables stakeholders to select according to their own normative priorities, preserving the value judgements that Muir et al. [25] identify as irreducibly contextual while providing the structured quantitative foundation that multi-criteria deliberation requires. Second, unlike the equity impact plane framework of Cookson et al. [5], which assumes intervention effects are known and fixed, H-RL-MUSYA couples multi-objective evolutionary optimization with agent-based behavioral simulation to discover intervention portfolios whose equity–efficiency positions account for realistic population responses—including behavioral feedback, social diffusion, and cross-domain interactions that standard MCDA cannot capture. Together, these two contributions advance beyond the option-selection problem that conventional multi-criteria frameworks address, toward a more fundamental challenge: systematically generating options that are both Pareto-efficient and behaviorally grounded.

The finding that single-objective-optimized policies performed substantially worse on non-optimized dimensions challenges prevailing cost-effectiveness maximization paradigms in health economics. Standard approaches scalarize objectives through willingness-to-pay thresholds or disability weights, creating an illusion of technical objectivity while actually imposing normative preferences through seemingly neutral parameters. Our Pareto approach acknowledges that policy selection inherently involves value judgments about distributive justice—questions properly resolved through democratic deliberation rather than algorithmic optimization. The framework’s contribution is not discovering “the best” policy but mapping the achievable frontier, enabling informed value-based selection among characterized alternatives.

The appropriate relationship between AI-based optimization and public health governance is one of structured decision-support, not algorithmic determination. The elements excluded from the current objective function—disease severity rankings, clinical effectiveness of interventions, priority population weighting, underpinning health policies, ethical priorities, and political feasibility—are not incidental omissions but central features of responsible resource allocation. They cannot be modeled away; they must be deliberated by practitioners who combine technical evidence with contextual knowledge, community values, and democratic accountability. The contribution of H-RL-MUSYA is to enrich the evidence available for that deliberation by generating a larger and more systematically characterized set of allocation options than expert intuition alone can produce, and by making the equity–efficiency trade-off structure explicit and quantified. This framing aligns with established public health decision-making traditions in which tools such as extended cost-effectiveness analysis, multi-criteria decision analysis, and program budgeting and marginal analysis serve as structured inputs to deliberative governance processes rather than as autonomous decision systems.

### 5.5. Safe Reinforcement Learning and the Limits of Algorithmic Foresight

The necessity of three-layer safety architecture reveals fundamental epistemological limits to purely data-driven policy optimization. Kim et al. [7] introduced safety-aware deep reinforcement learning for nephrotoxic medication management, explicitly modeling adverse outcomes including acute kidney injury and septic shock through offline RL algorithms. Yan et al. [8] proposed offline guarded safe RL combining action-level regularization with state-trajectory constraints for medical treatment, addressing out-of-distribution risks where inappropriate generalization beyond clinical expertise could yield harmful recommendations. Both approaches demonstrate algorithmic safety mechanisms preventing physiological harm predictable from electronic health records through learned risk models.

Our population policy application extends safe RL to qualitatively different failure modes requiring hybrid human–AI oversight. Kim et al.’s [7] adverse outcomes (kidney injury, septic shock) manifest through measurable biomarkers with established causal pathways from medications to physiological states. In contrast, harmful population policies often fail through social, political, or ethical mechanisms invisible in quantitative data: resource over-concentration violating equity norms, implementation timelines exceeding organizational capacity, coercive interventions undermining autonomy, or political constraints preventing deployment regardless of predicted effectiveness. The substantial fraction of harmful policies detected exclusively through expert oversight represents risks outside the simulator’s ontology—failures requiring contextual knowledge, ethical reasoning, and political judgment that resist formalization in algorithmic constraints.

This finding challenges common framings that position human oversight as temporary scaffolding during AI system development, to be progressively removed as algorithms achieve superhuman performance. In complex sociotechnical domains, human expertise provides qualitatively different knowledge than pattern recognition in historical data. Experts understand social context, anticipate political dynamics, and reason about autonomy and dignity through frameworks not reducible to statistical patterns. The expert safety layer’s unique contributions—identifying infrastructure gaps, political infeasibility, and ethical concerns—represent irreducible human judgment, complementing rather than competing with algorithmic safety mechanisms.

The three-layer architecture’s effectiveness stems from recognizing that different safety risks require different epistemic approaches. Conservative policy updates (Layer 1) prevent algorithmic instability through mathematical constraints on policy gradients—a purely computational concern addressable through trust-region optimization. Worst-case performance guarantees (Layer 2) address parametric uncertainty in behavioral models through robust optimization over plausible parameter ranges—requiring domain knowledge to specify uncertainty sets but operating algorithmically within those bounds. Expert oversight (Layer 3) evaluates emergent policy properties—implementation feasibility, political acceptability, and unintended consequences—that cannot be reduced to constraints on model parameters or state-action spaces.

This layered defense-in-depth approach aligns with Mussi et al. [23] and Cocaul et al.’s [22] emphasis on deploying RL in safety-critical domains requiring harm prevention during exploration. However, where the existing safe RL literature focuses primarily on algorithmic mechanisms (constrained MDPs, shielding, offline learning from safe demonstrations), our framework demonstrates that high-stakes social applications require integrating algorithmic rigor with human judgment. The minimal efficiency cost for comprehensive safety coverage suggests this hybrid approach need not fundamentally compromise learning effectiveness—safety and performance represent complementary rather than competing objectives when appropriately designed.

The deeper implication concerns the role of human expertise in algorithmically augmented governance. Rather than positioning AI systems as gradually replacing human decision-makers, our framework demonstrates a productive division of labor: algorithms excel at systematic exploration of vast policy spaces and optimization under precisely specified constraints; humans provide contextual interpretation, ethical reasoning, and judgment about emergent properties. This suggests governance models where AI systems generate candidate policies through structured exploration while human committees provide safety certification and final selection—combining algorithmic thoroughness with human wisdom.

### 5.6. From Hierarchical Architecture to Emergent Coordination

The hierarchical multi-agent architecture’s performance advantage over single-agent and flat multi-agent baselines reveals insights about scalability and coordination in complex decision systems. Low and Zhou [15] comprehensively reviewed cooperative MARL for robotic systems, highlighting value decomposition networks and communication protocols enabling coordination but identifying persistent challenges in scalability and credit assignment. Wang et al. [16] proposed robust constrained cooperative MARL for self-driving vehicles, demonstrating resilience through mean-field theory-based training. Hady et al. [17] surveyed MARL applications in resource allocation across energy microgrids and IoT systems, documenting hierarchical trust-region frameworks and multi-agent deep deterministic policy gradient approaches.

These applications share focus on systems with predetermined coordination structures: UAV swarms follow formation protocols, autonomous vehicles coordinate through traffic rules, and microgrids balance loads through physical constraints. In contrast, health policy optimization lacks predetermined coordination blueprints—the relationships between nutrition, mental health, behavioral risk, and accident prevention emerge from population-level dynamics rather than engineered protocols. Our framework’s learned coordination patterns, where nutrition and mental health agents synchronize interventions through emergent message-passing strategies, demonstrate that hierarchical MARL can discover effective coordination structures from data when interaction rewards incentivize cooperation.

The critical architectural insight is that hierarchy serves dual purposes beyond computational decomposition. First, the strategic–tactical separation creates natural abstraction barriers enabling meta-controllers to reason about portfolio-level outcomes without tracking implementation details, while tactical agents optimize domain-specific execution without global state explosion. Second, hierarchy provides temporal decomposition: quarterly meta-controller updates align with policy planning cycles, while monthly tactical adjustments respond to emerging trends—matching decision frequency to information availability and change rates. This temporal–functional hierarchy mirrors organizational structures in successful bureaucracies, suggesting deep isomorphism between effective coordination in human institutions and multi-agent AI systems.

The ablation analysis revealing performance degradation when removing domain communication mechanisms validates that health domains exhibit genuine interdependencies requiring coordination beyond parallel optimization. This distinguishes population health from domains like video game playing where hierarchical RL succeeds primarily through temporal abstraction rather than cross-subsystem coordination. The learned coordination patterns—nutrition agents adjusting interventions based on mental health messages, behavioral risk agents modulating intensity based on accident rate signals—represent data-driven discovery of intervention complementarities that domain decomposition without communication would miss.

However, the framework’s success raises questions about scaling to larger numbers of agents or more complex coordination requirements. Our four-domain architecture remains interpretable and computationally tractable; extending to dozens of health domains or finer-grained population segmentation might encounter the scalability challenges Low and Zhou [15] identified in robotic swarms. Future work might explore hierarchical communication structures—domain clusters exchanging information through specialized coordination agents—or attention-based messaging where agents selectively attend to relevant cross-domain signals rather than broadcasting to all partners.

### 5.7. Explainability and the Social Contract of Algorithmic Governance

The explainability mechanisms’ contribution to stakeholder acceptance addresses critical barriers to deploying AI in democratic governance contexts. Silveira et al. [10] systematically reviewed multi-agent clinical decision-support systems, identifying limited explainability as a key obstacle to real-world deployment despite promising technical capabilities. Our framework demonstrates that explainability serves multiple distinct functions requiring different technical approaches: attention visualization reveals temporal prioritization in strategic planning, SHAP analysis quantifies feature importance for domain-specific decisions, decision tree extraction exposes learned policy rules, and counterfactual explanation communicates marginal impacts of resource allocation choices.

The finding that explainability mechanisms substantially improved stakeholder trust without meaningful performance sacrifice challenges machine learning’s traditional accuracy–interpretability trade-off. This trade-off assumes users care primarily about predictive performance, tolerating opacity in exchange for marginal accuracy gains. In governance contexts, the calculus reverses: stakeholders demand transparency-enabling accountability and democratic oversight, potentially accepting performance costs for interpretability benefits. Our minimal performance sacrifice for substantial trust improvement suggests this assumed trade-off may be less severe than conventional wisdom suggests—at least in policy domains where decisions involve interpretable structured choices (intervention types, budget allocations, target populations) rather than high-dimensional unstructured inputs (images, text, sensor streams).

The deeper insight concerns the social contract underlying algorithmic governance. When AI systems make recommendations affecting public welfare, citizens have legitimate demands for explanation, enabling democratic scrutiny and contestation. Black-box optimization, regardless of performance, fundamentally conflicts with participatory governance norms requiring that power be visible and decisions be justifiable to those affected. The framework’s explainability mechanisms—particularly counterfactual explanations quantifying marginal policy impacts—transform algorithmic recommendations from opaque directives into interpretable decision support, enabling informed deliberation.

This distinguishes population policy AI from clinical decision-support or autonomous systems where different governance norms apply. Individual medical treatment, while requiring informed consent, typically delegates technical decisions to credentialed professionals operating under legal and ethical constraints. Autonomous vehicles prioritize safety over explainability—passengers care that the system works, not necessarily understanding its operation. Population policy, however, involves collective decisions over public resources affecting millions, requiring transparency enabling democratic participation beyond professional gatekeeping.

The alignment between learned policy rules and expert heuristics—nutrition agents prioritizing fortification when malnutrition exceeds thresholds matching WHO guidelines, mental health agents discovering sequential screening-before-treatment strategies endorsed by clinicians—provides a different form of validation than predictive accuracy alone. This concordance suggests the RL framework discovered clinically and epidemiologically valid strategies rather than exploiting simulator artifacts or data regularities disconnected from domain knowledge. However, it also raises questions about the value of algorithmic optimization if learned policies merely rediscover existing expert knowledge. The answer lies in portfolio-level optimization and cross-domain coordination: while individual domain strategies matched expert intuition, the strategic resource allocation across domains and learned coordination mechanisms represented novel configurations experts had not systematically explored.

Deploying AI in public health resource allocation raises ethical obligations along three axes. Transparency: the attention, SHAP, and counterfactual mechanisms (Section 4.7) make each allocation rationale inspectable, a precondition for democratic scrutiny of decisions affecting public funds. Accountability: the framework is positioned as decision support under the existing Provincial Health Office mandate—the expert-oversight layer preserves a human locus of responsibility, and no allocation is enacted without committee certification. Fairness: equity is treated as a first-class optimization objective rather than a post hoc check, and subgroup analyses (income, urban/rural, age) confirmed balanced gains; nonetheless, fairness defined over measured subgroups cannot capture all morally relevant distinctions, and contestation channels for affected communities remain necessary.

Relative to conventional regional planning—expert deliberation supported by cost-effectiveness analysis (CEA) and epidemiological forecasting—the framework differs in two respects: it optimizes equity and cost-effectiveness jointly rather than sequentially (standard CEA appraises interventions one at a time against a willingness-to-pay threshold), and it endogenizes behavioral response rather than assuming fixed per capita effects. The empirical gain over the expert committee baseline (15.7% additional DALYs averted) quantifies this difference. In low- and middle-income contexts, however, the principal barriers are organizational rather than algorithmic: fragmented surveillance, limited data-engineering capacity, and contested budget authority. We therefore frame the contribution as augmenting, not replacing, existing planning machinery, with phased adoption contingent on data and governance readiness.

### 5.8. Limitations and Boundary Conditions of Simulation-Based Optimization

Several fundamental limitations constrain the framework’s applicability and warrant explicit acknowledgment in public health language. The most consequential limitation is the restricted decision framework: the model optimizes across three quantifiable objectives—DALYs averted, cost-effectiveness, and equity as measured by the Gini coefficient—while leaving outside the objective space the full range of criteria that govern responsible public health resource allocation in practice. These excluded dimensions include: (a) disease severity and urgency, which may justify prioritizing acute or catastrophic conditions even at a higher cost per DALY; (b) the clinical effectiveness and evidence quality of specific interventions, which determines whether DALYs projected by the simulator are achievable in deployment; (c) age group and population prioritization—the model does not explicitly weight investments in children under five, working-age adults, or older adults, despite these groups carrying substantially different disease burdens, long-term multiplier effects, and ethical claims; (d) alignment with underpinning health policies, including Thailand’s prevention-first agenda, mental health service strengthening priorities, and non-communicable disease management strategies, which may justify allocations that diverge from a purely DALY-maximizing frontier; (e) political and institutional feasibility, including budget cycle constraints, workforce capacity, procurement timelines, and regulatory requirements; and (f) community preferences and values that should be elicited through participatory processes rather than inferred from surveillance data. These limitations mean that the Pareto policies generated by H-RL-MUSYA represent a structured starting point for deliberation—an enriched option set for multi-criteria decision analysis—rather than implementation-ready recommendations. Public health practitioners using this framework must supplement model outputs with the full range of criteria above before committing to an allocation strategy. Acknowledging and communicating these boundaries transparently is essential to the responsible deployment of AI-based decision support in health governance.

Second, the framework’s alignment with current public health policy directions warrants careful consideration. Policy environments evolve: priorities shift from acute-care and disease-specific programs toward prevention, health promotion, and whole-of-society approaches to social determinants. Thailand’s 13th National Health Development Plan (2023–2027) explicitly prioritizes primary prevention, reduction in health inequities, mental health service strengthening, and investment in early-life interventions—a configuration that may differ substantially from the historical allocation patterns against which H-RL-MUSYA’s agent-based simulator was calibrated. A framework optimized on historical data risks encoding and amplifying past allocation biases rather than supporting the policy transitions that health authorities seek to make. Users should critically assess whether the model’s calibrated behavioral parameters and outcome projections reflect the policy directions they intend to pursue, or whether recalibration against more recent or policy-aligned data is required. This limitation reflects a broader epistemological constraint on simulation-based learning: policies are only as robust as the environments in which they were optimized, and those environments are themselves historical constructs that may not represent desired futures.

Third, the multi-objective optimization framework maps the Pareto frontier across three quantified dimensions but provides no principled method for selecting among non-dominated policies. This is philosophically appropriate—final selection requires value judgements properly made through democratic and deliberative processes rather than algorithmic optimization. However, it creates a practical implementation gap: without a structured multi-criteria decision analysis framework to bridge model outputs and allocation decisions, practitioners may default to selecting the DALY-maximizing option, inadvertently treating DALYs as a scalar metric despite the model’s explicit multi-objective design. Future implementations should provide structured decision-support tools—such as interactive Pareto visualizations with stakeholder weighting mechanisms, extended cost-effectiveness analysis overlays, or program budgeting and marginal analysis templates—to facilitate informed selection among non-dominated strategies.

Third, the safe reinforcement learning mechanisms, while preventing harmful policies during training, cannot guarantee safety under deployment. The three safety layers address risks visible within the simulator’s ontology (behavioral instability through KL constraints, parametric uncertainty through robust optimization, feasibility through expert review), but cannot anticipate truly novel failure modes outside the framework’s conceptual space. This reflects a general limitation of safety verification: we can prove systems are safe with respect to specified properties under stated assumptions, but cannot guarantee the absence of unspecified risks or assumption violations. The precautionary approach should involve gradual deployment with continuous monitoring, maintaining expert oversight during operational use rather than only during training, and developing mechanisms for rapid policy adjustment when unexpected consequences emerge.

Fourth, the framework’s computational requirements—hundreds of GPU-hours for training, large-scale agent-based simulation—may limit accessibility to well-resourced institutions, potentially exacerbating health equity at the meta-level even while optimizing equity within regions. This raises questions about technology transfer to resource-constrained settings where population health challenges are often most acute. Cloud computing and open-source implementation provide partial solutions, but fundamental barriers remain: data infrastructure requirements, technical capacity for model adaptation, and institutional readiness for algorithmic decision support. The framework’s value may lie not in universal deployment but in demonstrating methodological feasibility, enabling future work on more efficient architectures or simplified versions appropriate for diverse contexts.

Regarding scalability and feasibility, the current four-domain architecture trains in approximately 340 GPU-hours on a 1% population sample; computational cost scales approximately linearly with the number of domains and sub-linearly with the synthetic-agent count, so extension to additional domains is tractable, whereas finer population segmentation is the dominant cost driver. For resource-constrained deployment, the trained policy library is lightweight at inference and can be served on commodity hardware; the binding constraints are therefore not computation but data infrastructure (five years of multi-domain surveillance) and institutional capacity for expert oversight. A reduced-data, cloud-hosted configuration is outlined as a feasible entry path for lower-capacity settings.

### 5.9. Future Directions and the Research Frontier

Several promising directions emerge from this work’s findings and limitations. First, integrating causal inference frameworks could strengthen behavioral response modeling beyond correlational calibration. The agent-based simulator currently relies on approximate Bayesian computation matching observed trajectories, which captures correlations but may not identify true causal mechanisms. Instrumental variable methods exploiting natural experiments, regression discontinuity designs at policy thresholds, or synthetic control approaches could provide stronger causal identification of intervention effects. This would enhance simulator fidelity and enable more confident extrapolation to novel interventions lacking historical precedent.

Second, extending the framework to dynamic budget allocation across multi-year horizons could address sustainability concerns beyond quarterly optimization. Current implementation treats fiscal years independently, potentially selecting policies yielding short-term gains but long-term costs or creating path dependencies limiting future options. Multi-year planning with explicit intertemporal constraints could optimize sustainability while accounting for how current policies shape future feasibility. This might involve hierarchical RL with an additional temporal layer: strategic multi-year resource frameworks, operational annual budgets, and tactical quarterly implementations.

Third, incorporating climate change impacts, pandemic preparedness, and emerging health threats could enhance resilience to external shocks. The current framework optimizes for relatively stationary health challenges (nutritional deficiencies, mental health disorders, behavioral risks), but external disruptions—climate-driven disease emergence, economic crises, health system shocks—could fundamentally alter intervention effectiveness or priority rankings. Robust optimization across environmental scenarios or adversarial training against worst-case shocks might produce policies more resilient to unforeseen disruptions than those optimized under central tendency assumptions.

Fourth, developing federated learning protocols enabling privacy-preserving multi-region policy optimization could facilitate knowledge transfer while respecting data sovereignty. Current implementation requires centralizing health surveillance data from five provinces, raising privacy concerns and creating political barriers in contexts with stricter data protection requirements. Federated approaches—training local models on provincial data, aggregating policy insights without sharing individual records—could enable collaborative learning across regions or countries while maintaining data localization.

Fifth, randomized controlled trials comparing RL-optimized policies against expert-designed interventions in matched communities would provide gold-standard causal evidence of effectiveness. The prospective pilot demonstration employed quasi-experimental difference-in-differences analysis vulnerable to confounding from unmeasured provincial differences. Cluster randomization of districts to RL-optimized versus status quo policies would eliminate selection bias and enable definitive causal attribution, though ethical and practical considerations around withholding potentially beneficial interventions from control groups require careful navigation.

Sixth, exploring integration with participatory budgeting and citizen engagement processes could enhance democratic legitimacy beyond expert oversight. Current expert committee review ensures technical feasibility and ethical acceptability but involves professional gatekeepers rather than affected populations. Mechanisms enabling community input on value priorities, feedback on proposed interventions, or participatory selection among Pareto frontier alternatives could strengthen social license while surfacing local knowledge about implementation barriers or unintended consequences experts might miss.

Finally, theoretical work on sample efficiency and transfer learning could reduce data requirements, limiting applicability to regions with sparse surveillance infrastructure. The current framework requires five years of comprehensive health data for simulator calibration—a demanding requirement in contexts lacking established monitoring systems. Meta-learning approaches enabling transfer from data-rich to data-scarce regions, or techniques for policy optimization under greater uncertainty when calibration data are limited, could extend benefits to settings where population health optimization is most urgently needed but empirical foundations are weakest.

To function as an operational decision-support tool rather than a standalone model, H-RL-MUSYA is designed to interface with Thailand’s existing health information infrastructure. Inputs are ingested from the Ministry of Public Health Data Center (HDC) via its standardized 43-file export (and provincial JHCIS/HOSxP feeds), mapped to the simulator’s quarterly state schema through a documented ETL layer; budget and resource records are drawn from Provincial Health Office accounts. Outputs—the Pareto menu and the recommended portfolio with its SHAP and counterfactual explanations—are returned to planners as a quarterly briefing for committee certification, after which approved allocations are entered into the existing budget-execution workflow. No component bypasses the current governance chain; the framework occupies the appraisal step between data aggregation and committee decision.

### 5.10. Broader Implications for AI in Democratic Governance

This work’s significance extends beyond population health policy to fundamental questions about artificial intelligence’s role in democratic governance. The framework demonstrates that algorithmic systems can outperform traditional approaches in complex social optimization problems while maintaining transparency, safety, and alignment with democratic values. However, superior technical performance does not automatically confer legitimacy for algorithmic governance; that requires explicit attention to accountability, participation, and contestability.

The multi-objective optimization framework’s refusal to collapse competing values into scalar metrics represents one model for value-aligned AI in policy domains: instead of imposing algorithmic “solutions,” surface trade-offs enable human deliberation. The safe reinforcement learning mechanisms demonstrate that AI systems in high-stakes domains require hybrid architectures combining algorithmic thoroughness with human judgment rather than end-to-end automation. The explainability mechanisms show that technical performance must be balanced against interpretability, enabling democratic scrutiny.

These design principles—multi-objective transparency, human–AI collaboration, explainable recommendations—point toward governance models where AI augments rather than replaces democratic institutions. Algorithms excel at systematic exploration of vast possibility spaces and optimization under precisely specified constraints. Humans provide contextual interpretation, ethical reasoning, and final authority over value trade-offs. The productive division of labor combines algorithmic capabilities with human wisdom, creating decision-support systems that enhance rather than undermine democratic participation in collective choices affecting public welfare.

## 6. Conclusions

This study demonstrated that hierarchical multi-agent reinforcement learning, integrated with agent-based behavioral simulation and multi-objective Pareto optimization, can enrich public health resource allocation decision-making by systematically generating and evaluating a diverse menu of evidence-based allocation strategies. Applied to Thailand’s Health Region 10, H-RL-MUSYA identified 127 Pareto-efficient allocation strategies across four priority health domains—nutrition, mental health, behavioral risk, and accident prevention—yielding a representative compromise allocation that averted 847,293 DALYs (34.1% improvement over historical baselines), improved cost-effectiveness by 31.3%, and reduced the health equity Gini coefficient from 0.243 to 0.187. A 12-month prospective pilot confirmed a +23.1% improvement in composite health outcomes, with 91% of implementers reporting the framework as useful decision support. These results should be interpreted in their proper context: H-RL-MUSYA functions as a decision-support tool that surfaces non-intuitive allocation strategies and quantifies equity–efficiency trade-offs. It does not—and should not—substitute for the multi-criteria deliberative processes through which responsible public health resource allocation decisions are made. DALYs averted and cost-effectiveness ratios, while important indicators, represent partial evaluation criteria. Final allocation decisions must integrate disease severity priorities, clinical evidence quality, population-group prioritization (children under five, working-age adults, older adults), alignment with national health policy directions, ethical commitments, feasibility constraints, and community preferences—dimensions that require human expertise, contextual knowledge, and democratic accountability.

These findings carry direct implications for public health governance in resource-constrained settings. First, AI-assisted policy exploration can meaningfully expand the option set available to health authorities, surfacing allocation strategies that expert committees may not systematically explore within conventional deliberative timeframes. The 127 Pareto-efficient policies identified include configurations that expert review subsequently confirmed as feasible and evidence-aligned, suggesting genuine additive value beyond reinforcing existing expert intuitions. Second, H-RL-MUSYA’s Pareto frontier makes the equity–efficiency trade-off structure transparent and quantified, enabling more informed dialog between health authorities, program managers, and communities about the values and priorities that should guide allocation. This transparency is particularly important in contexts such as Health Region 10, where historical allocations have produced inequitable distributions of health gains across socioeconomic quintiles and demographic groups. Third, the prospective pilot’s 91% implementer acceptance rate suggests that AI-generated policy options can achieve stakeholder legitimacy when accompanied by transparent explanation of how recommendations were generated and when presented as structured options for deliberation rather than directives for implementation. Fourth, the framework’s alignment with Thailand’s current health policy priorities—prevention-first, mental health strengthening, and equity reduction—demonstrates that AI-based decision-support tools can be designed to reflect evolving policy agendas rather than optimizing within historical status quo boundaries.

From a public health equity perspective, the reduction in the Gini coefficient from 0.243 to 0.187 represents a meaningful improvement in distributional fairness of health gains across socioeconomic quintiles—a dimension that cost-effectiveness analysis alone systematically fails to capture [5,21]. Importantly, this equity improvement was achieved without sacrificing overall health impact, suggesting that the equity–efficiency trade-off is less severe in the Health Region 10 context than standard cost-effectiveness frameworks imply. However, equity in health outcomes across demographic groups—particularly between children, working-age adults, and older adults—was not explicitly optimized in the current framework and warrants dedicated attention in future implementations. Population-group-specific equity monitoring would strengthen the framework’s alignment with the ‘leave no one behind’ commitments embedded in Thailand’s health development agenda and in Sustainable Development Goal 3.

Several limitations should be acknowledged. The agent-based simulator, while calibrated against 4.87 million person-quarter observations, represents a 1% sample of the regional population, and behavioral heterogeneity parameters remain uncertain at the subdistrict level. Most importantly, the three-objective framework—health impact, cost-effectiveness, and equity—captures essential but partial dimensions of public health resource allocation. Disease severity, clinical evidence quality, age-group priorities, policy alignment, feasibility, and community values lie outside the model’s scope and must be integrated through deliberative processes. The framework is therefore most appropriately deployed as a structured option-generation tool within a broader multi-criteria decision analysis process, not as a standalone allocation system. External validation in additional health regions and governance contexts, and randomized evaluation of AI-assisted versus conventional allocation processes, represent essential next steps toward establishing the framework’s generalizability and real-world effectiveness.

## Figures and Tables

**Table 1:** Hyperparameter specifications for H-RL-MUSYA framework.

Component	Parameter	Value	Justification
Meta-Controller	Architecture	Transformer (6 layers, 8 heads)	Capture long-range temporal dependencies
	Hidden dimension	512	Balance expressiveness and computational cost
	Planning horizon	Quarterly (20 steps/episode)	Align with policy decision cycles
Domain Agents	Architecture	Fully connected (3 layers)	Domain-specific feature extraction
	Hidden units	[256, 128, 64]	Progressive dimensionality reduction
	Message dimension	64	Inter-agent communication bandwidth
Population Simulator	Synthetic agents	460,000 (1% sample)	Computational feasibility with statistical validity
	Network density	0.08 (avg. degree 36,800)	Reflect observed social connectivity
	Utility parameters	α, β, γ, δ, ε ∈ [0, 1]	Calibrated via ABC (top 1000/100,000)
Multi-Objective Optimization	Policy population	100	Pareto frontier diversity
	Crossover probability	0.7	Standard evolutionary parameter
	Mutation rate	0.1	Exploration–exploitation balance
	Objective weights	Equal (1/3, 1/3, 1/3)	Unbiased multi-objective search
Safe RL	KL divergence threshold δ_safe_	0.01	Conservative constraint (10× stricter than standard)
	Uncertainty set size |U|	50 scenarios	Cover plausible behavioral variations
	Baseline performance V_baseline_	Historical policy mean	Ensure improvement guarantee
Training Configuration	Total episodes	2000	Empirical convergence threshold
	Episode length	5 years (20 quarters)	Policy evaluation horizon
	Discount factor γ	0.98	Value long-term health outcomes
	Learning rate (meta)	3 × 10^−4^	Adam optimizer with gradient clipping
	Learning rate (domain)	5 × 10^−4^	Faster tactical adaptation
	Batch size	32 episodes	Variance reduction in gradient estimates
	Convergence criterion	Hypervolume Δ < 0.1% (100 episodes)	Pareto frontier stabilization
Computational Resources	GPUs	8 × NVIDIA A100 (40GB)	Parallel episode simulation
	Training time	340 GPU-hours (42.5 h wall-clock)	Distributed training efficiency

**Table 2:** Baseline comparison matrix.

Method	Year	Multi-Obj	Hierarchical	Safety	ABM	Source
Status Quo	2019–2023	✗	✗	✗	✗	Regional data
Expert Committee	2024	△	✗	△	△	Workshop design
SAC	2018	✗	✗	✗	✓	ICML
DRL-OS-CMOEA	2024	✓	✗	✓	✗	IEEE/CAA JAS
SCPO-MARL	2024	✗	✓	✓	✓	NeurIPS
HC-RL-Framework	2025	△	△	△	△	Health Care Mgmt Sci.
MA-HealthCDS	2025	△	✓	△	△	Appl. Soft Comput.
H-RL-MUSYA	2025	✓	✓	✓	✓	This work

Note: ✓ = fully implemented, △ = partial/implicit implementation, ✗ = absent. ABM = Agent-Based Modeling.

**Table 3:** Comparative performance in simulated 5-year policy trials.

Method	DALYs Averted	Cost-Eff. (×10^3^)	Gini (Equity)	Cost (B THB)	Robust Perf.
Status Quo	631,842	34.5	0.243	18.3	0.731
Expert Committee	732,158	38.2	0.209	19.2	0.784
SAC	778,431	40.7	0.278	19.1	0.695
DRL-OS-CMOEA	724,893	41.3	0.251	17.6	0.742
SCPO-MARL	795,847	42.1	0.183	18.9	0.806
HC-RL-Framework	758,234	39.6	0.218	19.1	0.767
MA-HealthCDS	781,562	40.8	0.192	19.2	0.779
H-RL-MUSYA	847,293	45.3	0.187	18.7	0.823

Note: Cost-effectiveness measured as DALYs per million THB. Gini coefficient measures inequality (lower indicates greater equity). Robust performance shows worst-case normalized returns across uncertainty scenarios. All H-RL-MUSYA improvements significant at *p* < 0.001 unless otherwise noted.

**Table 4:** Retrospective validation against natural experiments.

Intervention	Observed Outcome	H-RL Predicted	Expert Predicted	Epidemiol. Model	H-RL % Error
Sodium Reduction (Ubon Ratchathani)	14.2% ↓ intake	13.1% ↓	11.8% ↓	10.3% ↓	7.7%
Alcohol Enforcement (Yasothon)	34.0% ↓ accidents	31.2% ↓	42.1% ↓	28.7% ↓	8.2%
Mental Health Expansion (Amnat Charoen)	16.0% ↑ screening	18.7% ↑	21.3% ↑	19.4% ↑	16.9%
Mean Absolute % Error	---	12.4%	15.2%	18.7%	---

Note: ↓ indicates reduction in negative outcomes; ↑ indicates improvement in positive outcomes. Predictions generated using pre-intervention data only (2019–2021 training window).

**Table 5:** Prospective pilot outcomes (12-month implementation).

Outcome Measure	Yasothon (Intervention)	Roi Et (Control)	DiD Estimate	*p*-Value
Primary Outcome				
Composite Health Score	+23.1 points	+2.4 points	+20.7 (18.7, 25.5)	<0.001
Domain-Specific Outcomes				
Nutrition Indicators	+19.4%	+3.1%	+16.3% (12.8, 19.7)	<0.001
Mental Health Screening	+31.8%	+5.2%	+26.6% (22.4, 30.8)	<0.001
Behavioral Risk Reduction	+16.2%	+2.8%	+13.4% (10.1, 16.7)	<0.001
Accident Rate Decline	−12.7%	−3.4%	−9.3% (−12.1, −6.5)	<0.001
Cost-Effectiveness & Equity				
DALYs per Million THB	42.8	34.1	+8.7 (6.2, 11.3)	<0.001
Gini Coefficient (Equity)	0.203	0.261	−0.058 (−0.073, −0.043)	<0.001

Note: DiD = Difference-in-differences controlling for baseline trends, population demographics, and temporal shocks; 95% confidence intervals in parentheses. Positive values indicate improvement except Gini (lower is better).

**Table 6:** Implementation fidelity analysis.

Factor	Simulated Assumption	Observed Reality	Impact on Performance
Program Initiation	Immediate (week 0)	Delayed 3.2 weeks (SD: 1.8)	−1.2% DALYs
Staff Training	Pre-trained	80 h per domain required	−1.8% DALYs
Compliance Rates	67% (ABM-calibrated)	63% (political/cultural adaptation)	−1.4% DALYs
Infrastructure Access	Universal	Limited rural connectivity	−1.1% DALYs
Total Deviation	100% fidelity	94.5% effective fidelity	−5.5% DALYs

Note: Impact estimated via counterfactual simulation adjusting parameters to observed values.

**Table 7:** Implementation among facilitators and barriers.

Category	Factor	Endorsement	Representative Quote
Facilitators			
Decision Support	Pareto frontier presentation	97%	“Transparent trade-offs enabled values-based selection acceptable to all stakeholders”
Adaptability	Quarterly meta-controller updates	94%	“Responsive to emerging dengue outbreak—updated priorities within 6 weeks”
Organizational Fit	Domain specialization	88%	“Matches our departmental structure—each team owns their agent’s domain”
Barriers			
Initial Trust	Algorithmic recommendation resistance	47% initial	“Resolved through expert oversight—now seen as decision support, not replacement”
Technical Capacity	Rural computational infrastructure	35% concern	“Cloud deployment solved—no local GPU requirements”
Data Quality	Incomplete district-level records	29% concern	“Improving—established data governance protocols”

Note: Endorsement measured as % reporting factor as “important” or “very important” in structured interviews.

**Table 8:** Ablation study results.

Model Variant	DALYs Averted	Cost-Eff.	Gini	Safety Violations
H-RL-MUSYA (Full)	847,293	45.3	0.187	0
w/o Hierarchical	778,431 (−8.1%)	40.7	0.278	3
w/o Agent-Based Modeling	726,114 (−14.3%)	38.8	0.198	2
w/o Multi-Objective	821,457 (−3.1%)	41.2	0.314	1
w/o Safe RL Mechanisms	867,142 (+2.3%)	46.4	0.173	47
w/o Domain Communication	803,527 (−5.2%)	43.0	0.201	0

Note: Safety violations indicate policies violating worst-case performance guarantees or expert safety criteria during training. Percentages show relative change from full model.

**Table 9:** Three-layer safety framework performance.

Safety Layer	Mechanism	Threshold	Rejected	% of Total	Primary Function
Layer 1	Conservative Updates	D_KL ≤ 0.01	18	38.3%	Prevent drastic behavioral shifts
Layer 2	Worst-Case Guarantees	V_robust > V_baseline	12	25.5%	Filter catastrophic failures under uncertainty
Layer 3	Expert Oversight	15-member committee	17	36.2%	Catch ethical, political, implementation risks
Total	Multi-Layer Defense	Combined	47	100%	Comprehensive safety coverage

Note: Percentages represent proportion of rejected policies. Total candidate policies: 2000.

**Table 10:** Rejected policy failure modes and detection patterns.

Failure Mode	Count	% of Rejected	Primary Detection Layer(s)	Example Violation
Resource Over-Concentration	11	23.4%	Layer 1 + Layer 3	>70% budget to single domain, Gini > 0.35
Aggressive Compliance Assumptions	9	19.1%	Layer 2	Assumed 85% uptake vs. 63% historical
Unrealistic Timelines	8	17.0%	Layer 3	Procurement exceeding 18-month capacity
Infrastructure Gaps	6	12.8%	Layer 3	Rural clinics lacking required equipment
Ethical Concerns	7	14.9%	Layer 3	Coercive behavioral interventions
Political Infeasibility	6	12.8%	Layer 3	Conflicting provincial mandates

Note: 47 total rejections across all modes.

**Table 11:** Domain agent decision patterns and expert alignment.

Domain	Top Policy Strategy	Triggering Conditions	Expert Agreement	Example Rationale
Nutrition	Universal fortification	Malnutrition prevalence > 15%	94%	“Prioritize school feeding when child stunting exceeds threshold”
Mental Health	Targeted screening + CBT	Depression rate > 8%, PHQ-9 access < 60%	89%	“Expand screening capacity before intensive treatment programs”
Behavioral Risk	Community-based campaigns	Smoking rate > 25%, low enforcement	87%	“Social norm interventions more effective than taxation alone”
Accidents	Infrastructure + enforcement	Accident hotspots (>5 deaths/year)	92%	“Combined engineering and behavioral interventions for high-risk zones”

Note: Expert agreement measured via 15-member committee review of 50 randomly sampled policy recommendations. CBT = Cognitive Behavioral Therapy; PHQ-9 = Patient Health Questionnaire-9.

## Data Availability

The data presented in this study are available upon request from the corresponding author.
